# Supplementary material for: The nonequilibrium cost of accurate information processing
Source: Nat Commun. 2022 Nov 22;13:7155. doi: 10.1038/s41467-022-34541-w (PMC9684527; doi:10.1038/s41467-022-34541-w)
Supplement: Supplementary file 1 — Supplementary Information [file 41467_2022_34541_MOESM1_ESM.pdf]

Supplementary Information: The nonequilibrium cost of accurate information processing

Giulio Chiribella*[1,2,3], Fei Meng[1,4], Renato Renner[5], and Man-Hong Yung[4,6]
[1]QICI Quantum Information and Computation Initiative,
Department of Computer Science, The University of Hong Kong, Pokfulam Road, Hong Kong SAR, China
[2]Department of Computer Science, University of Oxford, Parks Road, Oxford OX1 3QG, UK
[3]Perimeter Institute for Theoretical Physics, 31 Caroline St North, Waterloo, ON N2L 2Y5, Canada
[4]Department of Physics, Southern University of Science and Technology, Shenzhen 518055, China
[5]Institute for Theoretical Physics, ETH Zürich
[6]Shenzhen Key Laboratory of Quantum Science and Engineering, Shenzhen 518055, China
*giulio@cs.hku.hk

SUPPLEMENTARY NOTE 1: DERIVATION OF THE ACCURACY-NONEQUILIBRIUM TRADEOFF

In this note we establish a fundamental tradeoff between accuracy and nonequilibrium in the execution of any given information processing task. The note consists of three parts. In the first part, we derive a closed-form expression for the nonequilibrium cost of a given quantum channel, building on results by Faist and Renner [1]. In the second part, we derive the optimal tradeoff curve as a semidefinite program. Finally, in the third part we introduce a further constraint in the semidefinite program, which leads to the notion of reverse entropy and to the bound (5) in the main text.

The nonequilibrium cost of a given information processing task

Here we discuss the basic settings and definitions used in our paper to evaluate the nonequilibrium of cost of a given information processing task.

Let us start from the related problem of evaluating the cost of a specific quantum channel $\mathcal{M}$. The minimum number of clean qubits needed to realise a channel $\mathcal{M}$ using Gibbs preserving operations was derived by Faist and Renner in Ref. [1]. They considered realisations where the machine reproduces the action of the desired channel on a part of a given entangled state $|\Psi\rangle \in \mathcal{H}_A \otimes \mathcal{H}_R$, involving the input system A and a reference system R. For approximate realisations with error ϵ, the minimum number of clean qubits, called the *nonequilibrium cost* in our paper, was shown to be

$$c_\epsilon(\mathcal{M}, |\Psi\rangle) = - \max_{\substack{\mathcal{M}'(\Gamma_A) \ \le 2^{-\lambda}\Gamma_B \\ (\mathcal{M}' \otimes \mathcal{I}_R)(|\Psi\rangle\langle\Psi|) \ \approx_\epsilon (\mathcal{M} \otimes \mathcal{I}_R)(|\Psi\rangle\langle\Psi|)}} \lambda \,, \tag{1}$$

where the maximisation is over all quantum operations (completely positive trace non-increasing maps) $\mathcal{M}'$ with input system A and output system B, and $\approx_\epsilon$ denotes approximate equality with respect to a suitable distance measure.

In the following, we will set $\epsilon = 0$ and we will minimise the cost $c_0(\mathcal{M}, |\Psi\rangle)$ over all channels $\mathcal{M}$ that achieve a desired level of accuracy in a given information processing task $\mathcal{T}$. Specifically, we will evaluate the minimum cost

$$c_\mathcal{T}(F) := \min\{c_0(\mathcal{M}, |\Psi\rangle) \mid \mathcal{F}_\mathcal{T}(\mathcal{M}) \ge F\} \,, \tag{2}$$

where $\mathcal{F}_\mathcal{T}(\mathcal{M})$ is the accuracy measure (as defined in the main text) and F is the desired level of accuracy.

Here, the restriction to exact realisations ($\epsilon = 0$) is done without loss of generality, because the approximate implementation of the task $\mathcal{T}$ is already taken into account by the accuracy measure $\mathcal{F}_\mathcal{T}(\mathcal{M})$.

It is worth stressing that our approach is different from the approach of most works in the literature, where one fixes a channel $\mathcal{M}$ and asks what is the cost of implementing some ϵ-approximation of $\mathcal{M}$. Following this approach, one might be tempted to pick a channel $\mathcal{M}$ that implements the task $\mathcal{T}$ perfectly, and then to minimise the cost over all channels in an ϵ-neighbourhood of $\mathcal{M}$. This approach, however, would not work in general. The problem is that the correspondence between information processing tasks and quantum channels is generally not one-to-one. First, there exist tasks that cannot be implemented perfectly by any channel $\mathcal{M}$, such as, for example, ideal quantum cloning. Second, there exist tasks that can be implemented perfectly by more than one channel. In those cases, the minimum cost in an ϵ-neighbourhood of a specific channel $\mathcal{M}$ picked among those that achieve the task $\mathcal{T}$ perfectly may not be

equal to the minimum cost over all channels that achieve the task with error at most ϵ. This point is made clear by the following example. Consider the task of transforming the maximally mixed qubit state $\rho = I/2$ into the Gibbs state $\rho' = \Gamma$, for $\Gamma = 2/3\,|0\rangle\langle 0| + 1/3\,|1\rangle\langle 1|$. This task is achieved perfectly by the channel $\mathcal{M}_{\rm Gibbs}$ that maps every state into the Gibbs state. Channel $\mathcal{M}_{\rm Gibbs}$ is a Gibbs preserving operation and, as such, it has zero cost. Hence, the cost of the state transition $I/2 \mapsto \Gamma$ is zero. On the other hand, the state transition $I/d \mapsto \Gamma$ can also be perfectly achieved by any channel $\mathcal{M}$ satisfying the relations $\mathcal{M}(|0\rangle\langle 0|) = 2/3\,|1\rangle\langle 1| + 1/3\,|0\rangle\langle 0|$ and $\mathcal{M}(|1\rangle\langle 1|) = |0\rangle\langle 0|$. Any such channel $\mathcal{M}$ is not Gibbs preserving and therefore has a strictly positive cost. Hence, if we just pick channel $\mathcal{M}$ and ask what is the cost of ϵ-approximating it, we will generally get a positive cost, despite the fact that the cost of the state transition $I/2 \to \Gamma$ is zero.

Instead of minimising the cost over all channels in an ϵ-neighbourhood of a specific channel, here we minimise the cost over all physical implementations that achieve a accuracy F in the task $\mathcal{T}$. In this approach, the relevant minimisation problem is the one in Eq. (2). Indeed, every implementation of the task $\mathcal{T}$ realises—by definition without error—a specific channel $\mathcal{M}$. The minimum cost over all implementations that realise channel $\mathcal{M}$ without error is $c_0(\mathcal{M}, |\Psi\rangle)$. Hence, our goal is to minimise $c_0(\mathcal{M}, |\Psi\rangle)$ over all channels $\mathcal{M}$ that achieve accuracy F. These settings correspond to the minimisation problem in Eq. (2).

Let us now discuss the choice of the state $|\Psi\rangle$ in Eq. (2). For $\epsilon = 0$, the condition $(\mathcal{M}' \otimes \mathcal{I}_R)(|\Psi\rangle\langle\Psi|) = (\mathcal{M} \otimes \mathcal{I}_R)(|\Psi\rangle\langle\Psi|)$ in Eq. (1) is equivalent to

$$\mathcal{M}'(\Pi_A \rho \Pi_A) = \mathcal{M}(\Pi_A \rho \Pi_A)\,, \qquad \forall \rho \in \mathsf{St}(\mathcal{H}_A)\,, \tag{3}$$

where Π_A is the projector on the support of the marginal state $\rho_A := \mathrm{Tr}_R[\Psi\rangle\langle\Psi|]$. Hence, the cost does not depend specifically on the state $|\Psi\rangle$, but only on the projector Π_A. From now on, we will denote by

$$c(\mathcal{M}, \Pi_A) := c_{\epsilon=0}(\mathcal{M}, |\Psi\rangle) \tag{4}$$

the nonequilibrium cost that has to be paid for an exact realisation of the channel $\mathcal{M}$ upon input states in the support of Π_A.

With this notation, we have the following:

Proposition 1. *The nonequilibrium cost of a channel $\mathcal{M}$ upon inputs in the support of Π_A is upper bounded as*

$$c(\mathcal{M}, \Pi_A) \leq D_{\max}(\mathcal{M}(\Pi_A \Gamma_A \Pi_A) \| \Gamma_B)\,, \tag{5}$$

where $D_{\max}(\rho\|\sigma) := \log \left\| \sigma^{-\frac{1}{2}} \rho \sigma^{-\frac{1}{2}} \right\|$ is the max relative entropy [2]. When the support of Π_A is invariant under the group of time translations $U_t := e^{-itH_A/\hbar}$, $t \in \mathbb{R}$ (equivalently, when $[\Pi_A, H_A] = 0$), the equality sign holds and one has

$$c(\mathcal{M}, \Pi_A) = D_{\max}(\mathcal{M}(\Pi_A \Gamma_A \Pi_A) \| \Gamma_B)\,. \tag{6}$$

Proof. For $\epsilon = 0$, Eq. (1) reads

$$c(\mathcal{M}, \Pi_A) = \min_{\mathcal{M}'(\Pi_A \rho \Pi_A) \;=\; \mathcal{M}(\Pi_A \rho \Pi_A)\,,\, \forall \rho \in \mathsf{St}(A)} \log \left\| \Gamma_B^{-\frac{1}{2}} \mathcal{M}'(\Gamma_A) \Gamma_B^{-\frac{1}{2}} \right\| \tag{7}$$

Choosing the quantum operation $\mathcal{M}'$ defined by $\mathcal{M}'(\rho) := \mathcal{M}(\Pi_A \rho \Pi_A)$, Eq. (7) yields the inequality $c(\mathcal{M}, \Pi_A) \leq \log \left\| \Gamma_B^{-\frac{1}{2}} \mathcal{M}(\Pi_A \rho \Pi_A) \Gamma_B^{-\frac{1}{2}} \right\| \equiv D_{\max}(\mathcal{M}(\Pi_A \Gamma_A \Pi_A) \| \Gamma_B)$, thus proving Eq. (5).

If the support of Π_A is time-invariant, then Π_A commutes with the Hamiltonian of system A and the Gibbs state can be written as $\Gamma_A = \Pi_A \Gamma_A \Pi_A + (I_A - \Pi_A)\,\Gamma_A\,(I_A - \Pi_A)$. Hence, we have the bound

$$\begin{aligned} \left\| \Gamma_B^{-\frac{1}{2}} \mathcal{M}'(\Gamma_A) \Gamma_B^{-\frac{1}{2}} \right\| &= \left\| \Gamma_B^{-\frac{1}{2}} \mathcal{M}'(\Pi_A \Gamma_A \Pi_A) \Gamma_B^{-\frac{1}{2}} + \Gamma_B^{-\frac{1}{2}} \mathcal{M}'((I_A - \Pi_A) \Gamma_A (I_A - \Pi_A)) \Gamma_B^{-\frac{1}{2}} \right\| \\ &\geq \left\| \Gamma_B^{-\frac{1}{2}}\, \mathcal{M}'(\Pi_A \Gamma_A \Pi_A)\, \Gamma_B^{-\frac{1}{2}} \right\| \\ &= \left\| \Gamma_B^{-\frac{1}{2}} \mathcal{M}(\Pi_A \Gamma_A \Pi_A) \Gamma_B^{-\frac{1}{2}} \right\|\,, \end{aligned} \tag{8}$$

where the inequality is due to the relation $\|A + B\| \geq \|A\|$ valid for arbitrary positive operators A and B, and the second equality is due to the condition (3). Taking the logarithm on both sides, and minimising over $\mathcal{M}'$, we obtain the inequality $c(\mathcal{M}, \Pi_A) \geq D_{\max}(\mathcal{M}(\Pi_A \Gamma_A \Pi_A) \| \Gamma_B)$. $\square$

Exact expression for the accuracy-nonequilibrium tradeoff

Here we provide an exact expression for the amount of nonequilibrium needed to achieve a desired level of accuracy in a given task.

The problem is to find the quantum channel (completely positive trace-preserving map) $\mathcal{M}$ that has minimum nonequilibrium cost among all the channels that attain accuracy at least F. To get started, we recall the expression for accuracy of a channel, defined in terms of a general performance test, as in the Methods section. For a task $\mathcal{T}$ defined by a set of input states $(\rho_x)_{x\in\mathsf{X}}$ and a set of output observables $(O_x)_{x\in\mathsf{X}}$, the worst-case accuracy of the channel $\mathcal{M}$ is defined as $\mathcal{F}_{\mathcal{T}}(\mathcal{M}) := \min_x \operatorname{Tr}[O_x\mathcal{M}(\rho_x)]$. In the Choi representation, the accuracy can be expressed as $\mathcal{F}_{\mathcal{T}}(\mathcal{M}) = \min_x \operatorname{Tr}[M\Omega_x]$, with $\Omega_x = \rho_x^T \otimes O_x$. In general, a performance test $\mathcal{T}$ is specified by a set of performance operators $(\Omega_x)_{x\in\mathsf{X}}$ and the accuracy is given by $\mathcal{F}_{\mathcal{T}}(\mathcal{M}) = \min_x \operatorname{Tr}[M\Omega_x]$.

Now, consider the average accuracy with respect to a probability distribution $\mathbf{p} = (p_x)_{x\in\mathsf{X}}$, defined as

$$\mathcal{F}_{\mathcal{T},\mathbf{p}}(\mathcal{M}) := \operatorname{Tr}[M\,\Omega_{\mathbf{p}}]\,, \tag{9}$$

where $\Omega_{\mathbf{p}} := \sum_x p_x\,\Omega_x$ is the average performance operator associated to the given task. Note that the performance depends only on the projection of the Choi operator on the support of $\Omega_{\mathbf{p}}$. Defining the marginal operator $\omega_A := \operatorname{Tr}_B[\Omega_{\mathbf{p}}]$, the projector Π_A onto the support of ω_A, and the projected Choi operator $\widetilde{M} := (\Pi_A\otimes I_B)\,M\,(\Pi_A\otimes I_B)$, we have the relation $\mathcal{F}_{\mathbf{p}}(\mathcal{M}) = \operatorname{Tr}\left[\widetilde{M}\,\Omega_{\mathbf{p}}\right]$, meaning that the accuracy depends only on $\widetilde{M}$, rather than on the full operator M.

The nonequilibrium cost for the implementation of the channel $\mathcal{M}$ on the support of Π_A is given by Eq. (6). In turn, the norm in Eq. (6) can be expressed as

$$\left\|\Gamma_B^{-\frac{1}{2}}\,\mathcal{M}(\Pi_A\Gamma_A\Pi_A)\,\Gamma_B^{-\frac{1}{2}}\right\| = \min_{\mathcal{M}(\Pi_A\Gamma_A\Pi_A)\le\lambda\,\Gamma_B} \lambda\,. \tag{10}$$

Now, recall that the action of a quantum channel on a given operator ρ (not necessarily a quantum state) is given by $\mathcal{M}(\rho) = \operatorname{Tr}_A[(\rho^T\otimes I_A)\,M]$, where ρ^T denotes the transpose of ρ with respect to a fixed bases, here chosen to consist of energy eigenstates. Choosing $\rho = \Pi_A\Gamma_A\Pi_A$, we then obtain the equality

$$\mathcal{M}(\Pi_A\Gamma_A\Pi_A) = \operatorname{Tr}_A[(I_B\otimes\Pi_A\Gamma_A\Pi_A)\,M]\,, \tag{11}$$

where we used the fact that Γ_A and Π_A are diagonal in the energy eigenbasis, and therefore $\Gamma_A^T = \Gamma_A$ and $\Pi_A^T = \Pi_A$.

Hence, the minimum nonequilibrium cost for achieving average accuracy at least F, denoted by $c_{\mathcal{T},\mathbf{p}}(F)$, can be written as

$$\begin{aligned} c_{\mathcal{T},\mathbf{p}}(F) &= \min\{c(\mathcal{M},\Pi_A)\mid\mathcal{F}_{\mathcal{T},\mathbf{p}}(\mathcal{M})\ge F\}\\ &= \log \min_{\substack{M\ge0\,,\,\operatorname{Tr}_B[M]=I_A\\ \lambda\,\Gamma_B\ge\operatorname{Tr}_A[(\Pi_A\Gamma_A\Pi_A\otimes I_B)\,M]\\ \operatorname{Tr}[M\,\Omega_{\mathbf{p}}]\ge F}} \lambda\,. \end{aligned} \tag{12}$$

This minimisation problem is a semidefinite program and can be solved efficiently with existing software packages.

We now connect minimisation of the average cost to the minimisation of the worst case cost:

Proposition 2. *For every test $\mathcal{T}$, one has the equality $c_{\mathcal{T}}(F) = \max_{\mathbf{p}} c_{\mathcal{T},\mathbf{p}}(F)$ for every $F\in[F_{\min},F_{\max}]$.*

Proof. The inequality

$$c_{\mathcal{T}}(F) \ge \max_{\mathbf{p}} c_{\mathcal{T},\mathbf{p}}(F) \tag{13}$$

is immediate from the fact that the worst case accuracy cannot be larger than the average accuracy. We now show the converse inequality. To this purpose, we recall the definition

$$F_{\mathcal{T}}(c) := \max_{\mathcal{M}:\ c(\mathcal{M},\Pi_A)\le c} F_{\mathcal{T}}(\mathcal{M}) = \max_{\mathcal{M}:\ c(\mathcal{M},\Pi_A)\le c}\ \min_{\mathbf{p}} F_{\mathcal{T},\mathbf{p}}(\mathcal{M})\,. \tag{14}$$

and use the equality

$$
\begin{aligned}
F_{\mathcal{T}}(c) &= \max_{\mathcal{M}\colon\, c(\mathcal{M},\Pi_A)\le c}\ \min_{\mathbf{p}} F_{\mathcal{T},\mathbf{p}}(\mathcal{M}) \\
&= \max_{\substack{M\ge 0\,,\,\mathrm{Tr}_B[M]=I_A\\ \mathrm{Tr}_A[(\Pi_A\Gamma_A\Pi_A\otimes I_B)\,M]\le 2^c\,\Gamma_B}}\ \min_{\mathbf{p}}\ \mathrm{Tr}[M\,\Omega_{\mathbf{p}}] \\
&= \min_{\mathbf{p}}\ \max_{\substack{M\ge 0\,,\,\mathrm{Tr}_B[M]=I_A\\ \mathrm{Tr}_A[(\Pi_A\Gamma_A\Pi_A\otimes I_B)\,M]\le 2^c\,\Gamma_B}}\ \mathrm{Tr}[M\,\Omega_{\mathbf{p}}] \\
&= \min_{\mathbf{p}} F_{\mathcal{T},\mathbf{p}}(c)\,,
\end{aligned} \tag{15}
$$

where we defined

$$
F_{\mathcal{T},\mathbf{p}}(c) := \max_{\mathcal{M}\colon\, c(\mathcal{M},\Pi_A)\le c} F_{\mathcal{T},\mathbf{p}}(\mathcal{M})\,. \tag{16}
$$

The exchange of the minimum and maximum in Eq. (15) is possible thanks to von Neumann's minimax theorem.

For every fixed value of c, the equality $F_{\mathcal{T}}(c) = \min_{\mathbf{p}} F_{\mathcal{T},\mathbf{p}}(c)$ implies that there exists a probability distribution $\mathbf{p}_0$ such that $F_{\mathcal{T},\mathbf{p}_0}(c) = F_{\mathcal{T}}(c)$. Now, let $\mathcal{M}$ be an arbitrary channel such that $F_{\mathcal{T},\mathbf{p}_0}(\mathcal{M}) \ge F_{\mathcal{T}}(c) =: F$. By definition, the cost of this channel must satisfy $c(\mathcal{M},\Pi_A)\ge c$. Hence, we obtain

$$
c_{\mathcal{T},\mathbf{p}_0}(F) = \min_{\mathcal{M}\colon\, F_{\mathcal{T},\mathbf{p}_0}(\mathcal{M})\ge F} c(\mathcal{M},\Pi_A) \ge c\,. \tag{17}
$$

On the other hand, the definition $F := F_{\mathcal{T}}(c)$ implies $c = c_{\mathcal{T}}(F)$ for every $F\in[F_{\min},F_{\max}]$. Hence, we obtained the inequality $c_{\mathcal{T},\mathbf{p}_0}(F)\ge c_{\mathcal{T}}(F)$, and therefore

$$
\max_{\mathbf{p}} c_{\mathcal{T},\mathbf{p}}(F) \ge c_{\mathcal{T}}(F)\,. \tag{18}
$$

The thesis then follows from Eqs. (13) and (18). □

Summarising, we have derived the following expression for the nonequilibrium cost:

$$
c_{\mathcal{T}}(F) = \max_{\mathbf{p}} c_{\mathcal{T},\mathbf{p}}(F) = \log \max_{\mathbf{p}} \min_{\substack{M\ge 0\,,\,\mathrm{Tr}_B[M]=I_A\\ \lambda\,\Gamma_B\ge \mathrm{Tr}_A[(\Pi_A\Gamma_A\Pi_A\otimes I_B)\,M]\\ \mathrm{Tr}[M\,\Omega_{\mathbf{p}}]\ge F}} \lambda\,. \tag{19}
$$

Lower bound on the nonequilibrium cost

For every fixed probability distribution $\mathbf{p}$, the optimisation over M in Eq. (19) is a semidefinite program. This semidefinite program (12) admits a dual formulation (see *e.g.* [3] for the basics of the duality theory), which yields the bound

$$
\min_{\substack{M\ge 0\,,\,\mathrm{Tr}_B[M]=I_A\\ \lambda\,\Gamma_B\ge \mathrm{Tr}_A[(\Pi_A\Gamma_A\Pi_A\otimes I_B)\,M]\\ \mathrm{Tr}[M\,\Omega_{\mathbf{p}}]\ge F\,.}} \lambda \ \ge \max_{\substack{X_A\otimes I_B + z\,\Omega_{\mathbf{p}}\le \Pi_A\Gamma_A\Pi_A\otimes Y_B\\ \mathrm{Tr}[\Gamma_B Y_B]\le 1}} \mathrm{Tr}[X_A] + z\,F \tag{20}
$$

The inequality is in fact an equality, because the above program satisfies the condition of strong duality [3], although this fact will not be used in the following.

We now introduce a simplification in the dual program (20). The simplification consists in restricting the maximisation to triples (X_A, Y_B, z) where the operator X_A is set to zero. This constraint leads to the new maximisation problem, whose optimal value is below the optimal value on the right-hand-side of (20). Explicitly, one has

$$
\max_{\substack{X_A\otimes I_B + z\,\Omega_{\mathbf{p}}\le \Pi_A\Gamma_A\Pi_A\otimes I_B\\ \mathrm{Tr}[\Gamma_B Y]\le 1}} \mathrm{Tr}[X] + z\,F \ge F\left(\max_{\substack{z\,\Omega_{\mathbf{p}}\le \Pi_A\Gamma_A\Pi_A\otimes Y_B\\ \mathrm{Tr}[\Gamma_B Y_B]\le 1}} z\right)\,. \tag{21}
$$

The new maximisation problem admits a closed-form solution. To find it, we use the change of variables $\sigma_B := \Gamma_B^{1/2}\, Y_B\, \Gamma_B^{1/2}$. With this change of variable, the inequality $z\,\Omega_{\mathbf{p}} \le \Pi_A\Gamma_A\Pi_A \otimes Y_B$ becomes $z\,\Omega_{\mathbf{p}} \le \Pi_A\Gamma_A\Pi_A \otimes \Gamma_B^{-1/2}\sigma_B\Gamma_B^{-1/2}$. In turn, this inequality is equivalent to $(\Gamma_A\otimes\Gamma_B^{-1})^{1/2}\,\Omega_{\mathbf{p}}\,(\Gamma_A\otimes\Gamma_B^{-1})^{1/2} \le I_A\otimes Y_B/z$. Finally, defining the operator $\Lambda_B = \sigma_B/z$, we obtain the condition $I_A\otimes\Lambda_B \ge (\Gamma_A\otimes\Gamma_B^{-1})^{1/2}\,\Omega_{\mathbf{p}}\,(\Gamma_A\otimes\Gamma_B^{-1})^{1/2} =: \omega_{\mathcal{T},\mathbf{p}}$, where the subscript $\mathcal{T}$ stresses the dependence of the operator $\omega_{\mathcal{T},\mathbf{p}}$ on the task $\mathcal{T}$. Hence, we obtained the equality

$$\max_{\substack{z\,\Omega_{\mathcal{T},\mathbf{p}} \le \Pi_A\Gamma_A\Pi_A\otimes Y_B \\ \mathrm{Tr}[\Gamma_B Y_B]\le 1}} z = \left(\min_{I_A\otimes\Lambda_B \ge \omega_{\mathcal{T},\mathbf{p}}} \mathrm{Tr}[\Lambda_B]\right)^{-1} = 2^{H_{\min}(A|B)_{\omega_{\mathcal{T},\mathbf{p}}}}\,. \tag{22}$$

Combining Eqs. (12), (21), and (22), we obtain the bound

$$c_{\mathcal{T},\mathbf{p}}(F) \ge H_{\min}(A|B)_{\omega_{\mathcal{T},\mathbf{p}}} + \log F\,, \tag{23}$$

which follows from Eq. (6). Maximising the l.h.s. over the choice of probability distribution $\mathbf{p}$ and using Proposition 2 we then obtain

$$c_{\mathcal{T}}(F) \ge H_{\min}(A|B)_{\omega_{\mathcal{T},\mathbf{p}}} + \log F \qquad \forall \mathbf{p}\,. \tag{24}$$

Finally, we maximise the r.h.s. over all probability distributions, thus obtaining

$$c_{\mathcal{T}}(F) \ge \kappa_{\mathcal{T}} + \log F\,, \tag{25}$$

where $\kappa_{\mathcal{T}} = \max_{\mathbf{p}} H_{\min}(A|B)_{\omega_{\mathcal{T},\mathbf{p}}}$ is the reverse entropy defined in the main text.

Eq. (25) can be reformulated as a bound on the maximum accuracy achievable with a given amount of nonequilibrium resources, namely

$$F_{\mathcal{T}}(c) \le 2^{c-\kappa_{\mathcal{T}}}\,. \tag{26}$$

SUPPLEMENTARY NOTE 2: THERMODYNAMIC IRREVERSIBILITY OF CLASSICAL DETERMINISTIC COMPUTATIONS

For the computation of a function $f : A \to B, x \mapsto f(x)$, where A and B are two finite sets, one has

$$\begin{aligned}\omega_{\mathcal{T},\mathbf{p}} &= \sum_{x\in A} p_x\,\frac{g_B(f(x))}{g_A(x)}\,|x\rangle\langle x| \otimes |f(x)\rangle\langle f(x)| \\ &= \sum_{y\in f(A)} g_B(y)\left(\sum_{x: f(x)=y} \frac{p_x}{g_A(x)}\,|x\rangle\langle x|\right)\otimes |y\rangle\langle y|\,,\end{aligned} \tag{27}$$

where $g_A(x)$ and $g_B(y)$ are the Gibbs probability distributions for the input and the output, respectively. Hence, the condition $I_A\otimes\Lambda_B \ge \omega_{\mathcal{T},\mathbf{p}}$ is equivalent to

$$\langle y|\Lambda_B|y\rangle \ge g_B(y)\max_{x:f(x)=y}\frac{p_x}{g_A(x)} \qquad \forall y\in f(A)\,. \tag{28}$$

Choosing the operator Λ_B that achieves the equality, we obtain

$$H_{\min}(A|B)_{\omega_{\mathcal{T},\mathbf{p}}} = -\log\left(\sum_{y\in f(A)} g_B(y)\max_{x:f(x)=y}\frac{p_x}{g_A(x)}\right)\,. \tag{29}$$

Now, we need to maximise the above expression over all probability distributions $\mathbf{p} = (p_x)$. First, note that the maximum is attained by probability distributions satisfying the condition

$$\frac{p_x}{g_A(x)} = \frac{p_{x'}}{g_A(x')} \qquad \forall x, x' : f(x) = f(x')\,. \tag{30}$$

Second, we define the probabilities $q_y := \sum_{x:f(x)=y} p_x$ and $p_f(y) := \sum_{x:f(x)=y} g_A(y)$. With this notation, we have the equality

$$\begin{aligned}\max_{\mathbf{p}} H_{\min}(A|B)_{\omega_{\mathcal{T},\mathbf{p}}} &= \max_{\mathbf{q}} -\log\left(\sum_{y\in f(A)} g_B(y)\,\frac{q_y}{p_f(y)}\right)\\ &= -\log\left(\min_{y\in f(A)}\,\frac{g_B(y)}{p_f(y)}\right)\\ &= D_{\max}(p_f\|g_B)\,. \end{aligned}\tag{31}$$

Hence, the reverse entropy of the classical computation of f is

$$\kappa_f = D_{\max}(p_f\|g_B)\,. \tag{32}$$

SUPPLEMENTARY NOTE 3: PROOF OF THE ATTAINABILITY CRITERION

In this note, we prove the attainability criterion given in the main text: specifically, we show that, if the bound (25) is saturated for the maximum accuracy $F = F_{\max}$, then the bound is tight for all values of the accuracy in the interval $F \in [F_{\min}, F_{\max}]$.

A. Evaluation of $F_{\min}$

By definition, $F_{\min}$ is the maximum worst-case fidelity achieved by a physical process with minimum nonequilibrium cost on the input subspace, namely $F_{\min} := \mathcal{F}_{\mathcal{T}}(c_{\min})$ where

$$c_{\min} := \min_{\mathcal{M}} c(\mathcal{M}, \Pi_A) \tag{33}$$

is the minimum nonequilibrium cost over all possible quantum channels acting on the support of Π_A. The minimum nonequilibrium cost can be readily evaluated, as shown in the following lemma:

Lemma 1. *The mininimum nonequilibrium cost for the realisation of an quantum channel upon inputs in the support of a projector Π_A with $[\Pi_A, H_A] = 0$ is*

$$c_{\min} = \log \mathrm{Tr}[\Pi_A\,\Gamma_A]\,, \tag{34}$$

and it is achieved by every quantum channel $\mathcal{M}$ satisfying the condition

$$\mathcal{M}(\widetilde{\Gamma}_A) = \Gamma_B\,, \qquad \text{with} \qquad \widetilde{\Gamma}_A := \frac{\Pi_A\Gamma_A\Pi_A}{\mathrm{Tr}[\Pi_A\Gamma_A\Pi_A]}\,. \tag{35}$$

Proof. For an arbitrary channel $\mathcal{M}$, Eq. (6) yields the expression

$$\begin{aligned} c(\mathcal{M},\Pi_A) &= D_{\max}(\mathcal{M}(\Pi_A\Gamma_A\Pi_A)\|\Gamma_B)\\ &= \log\left\|\Gamma_B^{-\frac{1}{2}}\,\mathcal{M}(\Pi_A\,\Gamma_A\,\Pi_A)\,\Gamma_B^{-\frac{1}{2}}\right\|\\ &= \log\mathrm{Tr}[\Pi_A\,\Gamma_A\,\Pi_A] + \log\left\|\Gamma_B^{-\frac{1}{2}}\,\mathcal{M}(\widetilde{\Gamma}_A)\,\Gamma_B^{-\frac{1}{2}}\right\|\\ &= \log\mathrm{Tr}[\Pi_A\,\Gamma_A] + D_{\max}(\mathcal{M}(\widetilde{\Gamma}_A)\,\|\,\Gamma_B)\ . \end{aligned}\tag{36}$$

Since the max relative entropy is non-negative, the above expression implies the bound $c(\mathcal{M},\Pi_A) \geq \mathrm{Tr}[\Pi_A\,\Gamma_A]$, valid for every quantum channel $\mathcal{M}$, and the equality holds if and only if $D_{\max}(\mathcal{M}(\widetilde{\Gamma}_A)\,\|\,\Gamma_B) = 0$, that is, if and only if condition (35) is satisfied. □

We now consider the set of all channels with minimum nonequilibrium cost $c_{\min}$, and, among them, we search for the channel with maximum accuracy.

Lemma 2. *If the bound (25) is attainable for a given value $F = F_0$, then $F_{\min} = 2^{c_{\min}-\kappa_{\mathcal{T}}}$.*

Proof. Note that, in general one has the relation

$$F_{\min} := \max_{\mathcal{M}: c(\mathcal{M},\Pi_A)\le c_{\min}} \mathcal{F}_{\mathcal{T}}(\mathcal{M}) \equiv \max_{\mathcal{M}: c(\mathcal{M},\Pi_A)= c_{\min}} \mathcal{F}_{\mathcal{T}}(\mathcal{M}) \le \max_{\mathcal{M}: c(\mathcal{M},\Pi_A)= c_{\min}} \le 2^{c(\mathcal{M},\Pi_A)-\kappa_{\mathcal{T}}} = 2^{c_{\min}-\kappa_{\mathcal{T}}} =: F_* \,, \tag{37}$$

where the inequality follows from Eq. (26). Our goal is to show that the bound $F_{\min} \le F_*$ holds with the equality sign whenever the bound (25) is attainable for a given value $F = F_0$.

Let $\mathcal{M}_0$ be a quantum channel that achieves the bound (25) at $F = F_0$, namely

$$\mathcal{F}_{\mathcal{T}}(\mathcal{M}_0) = F_0 \qquad \text{and} \qquad c(\mathcal{M}_0,\Pi_A) = \kappa_{\mathcal{T}} + \log F_0 \,. \tag{38}$$

Let us consider the case $F_0 = F_*$. In this case, we have

$$2^{c(\mathcal{M}_0,\Pi_A)-\kappa_{\mathcal{T}}} = F_0 = F_* = 2^{c_{\min}-\kappa_{\mathcal{T}}} \,, \tag{39}$$

which implies $c(\mathcal{M}_0,\Pi_A) = c_{\min}$. Since $F_{\min}$ is the maximum accuracy achieved by quantum channels with cost $c_{\min}$, we conclude that $F_* = \mathcal{F}_{\mathcal{T}}(\mathcal{M}_0) \le F_{\min}$. Combined with the Eq. (37), this bound implies $F_* = F_{\min}$.

Now, suppose that $F_0 > f$. Consider the parametric family of quantum channels $\mathcal{M}_F$ of the form

$$\mathcal{M}_F := p_F\, \mathcal{M}_0 + (1-p_F)\, \chi_B \ \mathrm{Tr}_A \qquad F \in [F_{\min}, F_0] \,, \tag{40}$$

where χ_B is a fixed quantum state (to be determined later) and $p_F := F/F_0$.

Note that the accuracy of the channel $\mathcal{M}_F$ is at least F, as one has

$$\mathcal{F}_{\mathcal{T}}(\mathcal{M}_F) \ge p_F\, F_0 = F \tag{41}$$

(the bound following from the fact that the performance operators are nonnegative).

We now set F to F_*, and choose the state χ_B so that the channel $\mathcal{M}_{F_*}$ has minimum cost. The minimum cost condition (35) implies

$$\chi_B = \frac{\Gamma_B - p_{F_*}\, \mathcal{M}_0(\widetilde{\Gamma}_A)}{1 - p_{F_*}} \,. \tag{42}$$

This expression is well-defined because $p_{F_*} = F_*/F_0$ is strictly smaller than 1.

We now show that Eq. (42) defines a valid quantum state. It is immediate to see that the operator χ_B has unit trace. It remains to show that χ_B is positive semidefinite. To this purpose, note that the condition $\chi_B \ge 0$ is equivalent to $\Gamma_B \ge p_{F_*}\, \mathcal{M}_0(\widetilde{\Gamma}_A)$, which is equivalent to $I_B \ge p_{F_*}\, \Gamma_B^{-\frac{1}{2}}\, \mathcal{M}_0(\widetilde{\Gamma}_A)\, \Gamma_B^{-\frac{1}{2}}$. In turn, this condition is equivalent to

$$1 \ge p_{F_*} \left\| \Gamma_B^{-\frac{1}{2}}\, \mathcal{M}_0(\widetilde{\Gamma}_A)\, \Gamma_B^{-\frac{1}{2}} \right\| \,. \tag{43}$$

We now show that Eq. (43) is satisfied. Inserting the definition of the state $\widetilde{\Gamma}_A$ [Eq. (35)] into Eq. (43), we obtain

$$\begin{aligned} p_{F_*} \left\| \Gamma_B^{-\frac{1}{2}}\, \mathcal{M}_0(\widetilde{\Gamma}_A)\, \Gamma_B^{-\frac{1}{2}} \right\| &= \frac{p_{F_*}}{\mathrm{Tr}[\Pi_A\, \Gamma_A\, \Pi_A]} \left\| \Gamma_B^{-\frac{1}{2}}\, \mathcal{M}_0(\Pi_A\, \Gamma_A\, \Pi_A)\, \Gamma_B^{-\frac{1}{2}} \right\| \\ &= \frac{p_{F_*}\, 2^{c(\mathcal{M}_0,\Pi_A)}}{2^{c_{\min}}} \,, \end{aligned} \tag{44}$$

having used Eqs. (6) and (34). Then, inserting the definition $p_{F_*} := F_*/F_0 = 2^{c_{\min}-\kappa_{\mathcal{T}}}/F_0$ and the relation $2^{c(\mathcal{M}_0,\Pi_A)} = F_0 2^{\kappa_{\mathcal{T}}}$ (following from Eq. (38)) in the right hand side, we obtain

$$\begin{aligned} p_{F_*} \left\| \Gamma_B^{-\frac{1}{2}}\, \mathcal{M}_0(\widetilde{\Gamma}_A)\, \Gamma_B^{-\frac{1}{2}} \right\| &= \frac{2^{c_{\min}-\kappa_{\mathcal{T}}}}{F_0}\ \frac{F_0\, 2^{\kappa_{\mathcal{T}}}}{2^{c_{\min}}} \\ &= 1 \,, \end{aligned} \tag{45}$$

Hence, condition (43) is satisfied.

In summary, the operator χ_B is a valid quantum state, and therefore the map $\mathcal{M}_F$ defined in Eq. (40) is a valid quantum channel. In particular, $\mathcal{M}_{F_*}$ is a valid quantum channel. The nonequilibrium cost of $\mathcal{M}_{F_*}$ is $c_{\min}$ and its accuracy is at least F_*. Since $F_{\min}$ is the maximum accuracy achievable with $c_{\min}$ clean qubits, we have the inequality $F_{\min} \ge F_* = 2^{c_{\min}-\kappa_{\mathcal{T}}}$. On the other hand, Eq. (37) gives the bound $F_{\min} \le 2^{c_{\min}-\kappa_{\mathcal{T}}}$. Hence, we conclude that the equality $F_{\min} = 2^{c_{\min}-\kappa_{\mathcal{T}}}$ holds. □

Corollary 1. *If the input subspace is the whole Hilbert space (i.e. $\Pi_A = I_A$), then the equality $F_{\min} = F^{\rm rev}_{\max}$ holds, where $F^{\rm rev}_{\max}$ is the accuracy of the time-reversed task defined in the Methods section of the main text.*

Proof. By Lemma 2, the minimum fidelity is $F_{\min} = 2^{c_{\min}-\kappa_{\mathcal{T}}}$. If the input subspace is the whole Hilbert space, then the projector Π_A is the identity operator, and Lemma 1 yields $c_{\min} = 0$. Hence, $F_{\min} = 2^{-\kappa_{\mathcal{T}}}$. Recalling the definition of the reverse entropy $\kappa := \log(1/F^{\rm rev}_{\max})$, one finally obtains the equality $F_{\min} = F^{\rm rev}_{\max}$. □

Proof of the attainability criterion

Theorem 1. *If the bound (25) is attainable at $F = F_0$, then it is attainable for every $F \in [F_{\min}, F_0]$, with $F_{\min} = 2^{c_{\min}-\kappa_{\mathcal{T}}}$.*

Proof. Let $\mathcal{M}_0$ be the channel that saturates the bound (25) at $F = F_0$, and let $\mathcal{M}_F$ be the channel defined in Eq. (40). The nonequilibrium cost of $\mathcal{M}_F$, given by Eq. (6), is

$$\begin{aligned} c(\mathcal{M}_F, \Pi_A) &= \log \left\| \Gamma_B^{-\frac{1}{2}} \mathcal{M}_F(\Pi_A\, \Gamma_A\, \Pi_A) \Gamma_B^{-\frac{1}{2}} \right\| \\ &= \log \mathrm{Tr}[\Pi_A\, \Gamma_A] + \log \left\| \Gamma_B^{-\frac{1}{2}} \mathcal{M}_F(\widetilde{\Gamma}_A) \Gamma_B^{-\frac{1}{2}} \right\| , \end{aligned} \tag{46}$$

where $\widetilde{\Gamma}_A$ is defined as in Eq. (35).

Using the definition of $\mathcal{M}_F$, we obtain

$$\begin{aligned} \mathcal{M}_F(\widetilde{\Gamma}_A) &= p_F\, \mathcal{M}_0(\widetilde{\Gamma}_A) + (1-p_F)\, \chi_B \\ &= p_F\, \mathcal{M}_0(\widetilde{\Gamma}_A) + (1-p_F)\, \frac{\Gamma_B - p_{F_*}\, \mathcal{M}_0(\widetilde{\Gamma}_A)}{1-p_{F_*}} , \end{aligned} \tag{47}$$

the second equality following from Eq. (42). Rearranging the terms, we obtain

$$\mathcal{M}_F(\widetilde{\Gamma}_A) = \frac{1-p_F}{1-p_{F_*}}\, \Gamma_B + \frac{p_F - p_{F_*}}{1-p_{F_*}}\, \mathcal{M}_0(\widetilde{\Gamma}_A) , \tag{48}$$

and therefore,

$$\begin{aligned} \left\| \Gamma_B^{-\frac{1}{2}} \mathcal{M}_F(\widetilde{\Gamma}_A) \Gamma_B^{-\frac{1}{2}} \right\| &= \left\| \frac{1-p_F}{1-p_{F_*}}\, I_B + \frac{p_F - p_{F_*}}{1-p_{F_*}}\, \Gamma_B^{-\frac{1}{2}}\, \mathcal{M}_0(\widetilde{\Gamma}_A)\, \Gamma_B^{-\frac{1}{2}} \right\| \\ &= \frac{1-p_F}{1-p_{F_*}} + \frac{p_F - p_{F_*}}{1-p_{F_*}} \left\| \Gamma_B^{-\frac{1}{2}}\, \mathcal{M}_0(\widetilde{\Gamma}_A)\, \Gamma_B^{-\frac{1}{2}} \right\| \\ &= \frac{1-p_F}{1-p_{F_*}} + \frac{p_F - p_{F_*}}{1-p_{F_*}}\, \frac{\left\| \Gamma_B^{-\frac{1}{2}}\, \mathcal{M}_0(\Pi_A\, \Gamma_A\, \Pi_A)\, \Gamma_B^{-\frac{1}{2}} \right\|}{\mathrm{Tr}[\Pi_A\, \Gamma_A]} \\ &= \frac{1-p_F}{1-p_{F_*}} + \frac{p_F - p_{F_*}}{1-p_{F_*}}\, 2^{c(\mathcal{M}_0, \Pi_A) - c_{\min}} , \end{aligned} \tag{49}$$

where the last equation follows from Eqs. (34) and (6). Using Eq. (38) and the definition $F_* = 2^{c_{\min}-\kappa_{\mathcal{T}}}$, we then obtain

$$\begin{aligned} \left\| \Gamma_B^{-\frac{1}{2}} \mathcal{M}_F(\widetilde{\Gamma}_A) \Gamma_B^{-\frac{1}{2}} \right\| &= \frac{1-p_F}{1-p_{F_*}} + \frac{p_F - p_{F_*}}{1-p_{F_*}}\, \frac{F_0}{F_*} \\ &= \frac{1-p_F}{1-p_{F_*}} + \frac{p_F - p_{F_*}}{1-p_{F_*}}\, \frac{1}{p_{F_*}} \\ &= \frac{p_F}{p_{F_*}} \\ &= \frac{F}{F_*} . \end{aligned} \tag{50}$$

Inserting Eqs. (34) and (50) into Eq. (46), we finally obtain

$$\begin{aligned} c(\mathcal{M}_F, \Pi_A) &= c_{\min} + \log \frac{F}{F_*} \\ &= \kappa_{\mathcal{T}} + \log F , \end{aligned} \tag{51}$$

where the last equality follows from the definition $F_* := 2^{c_{\min} - \kappa_{\mathcal{T}}}$.

Summarising, the channel $\mathcal{M}_F$ has nonequilibrium cost $\kappa_{\mathcal{T}} + \log F$, and accuracy at least F (by Eq. (41)). To conclude, we observe that channel $\mathcal{M}_F$ has accuracy $\mathcal{F}_{\mathcal{T}}(\mathcal{M}_F) = F$. Indeed, we know from Eq. (41) that the accuracy satisfies the inequality $\mathcal{F}_{\mathcal{T}}(\mathcal{M}_F) \geq F$. On the other hand, the bound (25) implies the inequality

$$\begin{aligned} \kappa_{\mathcal{T}} + \log F &= c(\mathcal{M}_F, \Pi_A) \\ &\geq \kappa_{\mathcal{T}} + \log \mathcal{F}_{\mathcal{T}}(\mathcal{M}_F), \end{aligned} \tag{52}$$

which implies $\mathcal{F}_{\mathcal{T}}(\mathcal{M}_F) \leq F$.

Summarising, the channel $\mathcal{M}_F$ has nonequilibrium cost $\kappa_{\mathcal{T}} + \log F$, and accuracy F (by Eq. (41)). Hence, it saturates the bound (25). □

SUPPLEMENTARY NOTE 4: ATTAINABILITY RESULTS

Classical deterministic computations. Here we show that the bound (25) is attainable for every classical deterministic computation. Let $f : \{1, \dots, d_A\} \to \{1, \dots, d_B\}, x \mapsto y = f(x)$ be a function between two finite sets. Mathematically, a classical process that evaluates the function f without errors can be represented as a quantum channel $\mathcal{M}_f$, whose action on a generic input state ρ is given by

$$\mathcal{M}_f(\rho) = \sum_x \langle x|\rho|x\rangle \, |f(x)\rangle\langle f(x)| \, . \tag{53}$$

For an approximate realisation of erasure, corresponding to a different channel $\mathcal{M}$, we consider the fidelity $F_f(\mathcal{M}) = \min_x \langle f(x)|\mathcal{M}(|x\rangle\langle x|)|f(x)\rangle$ as the figure of merit. Operationally, the fidelity corresponds to the probability that the channel $\mathcal{M}$ computes the correct value of $f(x)$, in the worst case over all possible inputs x.

Proposition 3. *The nonequilibrium cost for approximately computing the function f with fidelity F is $c_f(F) = \kappa_f + \log F$, where $\kappa_f = D_{\max}(p_f \| g_B)$ is the reverse entropy derived in Supplementary Note 2. The equality holds for every F in the interval $[F_{\min}, F_{\max}]$, with $F_{\min} = 2^{c_{\min} - \kappa_f}$ and $F_{\max} = 1$.*

Proof. Applying the channel to the Gibbs state Γ_A, we obtain

$$\mathcal{M}_f(\Gamma_A) = \sum_{y \in f(A)} p_f(y) \, |y\rangle\langle y| \qquad p_f(y) := \sum_{x : f(x) = y} g_A(x) \, . \tag{54}$$

Hence, the nonequilibrium cost of channel $\mathcal{M}_f$, given by Eq. (6) is

$$c(\mathcal{M}_f, \Pi_A) = D_{\max}(\mathcal{M}(\Gamma_A) \| \Gamma_B) = D_{\max}(p_f \| g_B) \, . \tag{55}$$

Comparing this equation with Eq. (32), we obtain the equality

$$c(\mathcal{M}_f, \Pi_A) = \kappa_f \, , \tag{56}$$

which implies

$$c_f(1) \leq c(\mathcal{M}_f, \Pi_A) = \kappa_f \, , \tag{57}$$

because the channel $\mathcal{M}_f$ has unit accuracy.

On the other hand, evaluating the bound (25) at $F = 1$ yields the converse inequality

$$c_f(1) \geq \kappa_f \, . \tag{58}$$

Hence, the equality $c_f(1) = \kappa_f$ holds.

Summarising, the bound (25) holds at $F = 1$ with the equality sign. Hence, Theorem 1 implies that the bound (25) holds for every F in the interval $[F_{\min}, 1]$. □

Quantum extensions of classical computations. Here we show that the bound (25) is attainable for every quantum extension (in the sense defined precisely in the following) of a classical computation.

Let $f : \{1, \dots, d_A\} \to \{1, \dots, d_B\}, x \mapsto y = f(x)$ be a function between two finite sets, and let $\widehat{\mathcal{M}_f}$ be a quantum channel from a d_A-dimensional input system A to a d_B-dimensional output system B, satisfying the condition

$$\widehat{\mathcal{M}_f}(|x\rangle\langle x|) = |f(x)\rangle\langle f(x)| \qquad \forall x \in \{1, \dots, d_A\} \, . \tag{59}$$

We call the channel $\widehat{\mathcal{M}}_f$ a *quantum extension* of the function f.

Now, consider the task of transforming the input state ρ_x into the target output state $\rho'_x = \mathcal{M}_f(\rho_x)$, where the input state ρ_x is picked from a set that includes the classical states $\{|x\rangle\langle x|\}_{x=1}^{d_A}$. When the output states ρ'_x are mixed, we assume that the figure of merit $\widehat{\mathcal{F}}_f$ for the state transformation task $\rho_x \mapsto \widehat{\mathcal{M}}_f(\rho_x)$ has been chosen in such a way that the channel $\widehat{\mathcal{M}}_f$ has accuracy $\widehat{\mathcal{F}}_f(\widehat{\mathcal{M}}_f) = 1$. When these conditions are satisfied, we call the state transformation task $\rho_x \mapsto \widehat{\mathcal{M}}_f(\rho_x)$ a quantum extension of the classical task of computing the function f.

Proposition 4. *Let $\widehat{\mathcal{M}}_f$ be a quantum extension of the function f, and let the state transformation task $\rho_x \mapsto \widehat{\mathcal{M}}_F(\rho_x)$ be a quantum extension of the task of computing the function f. The quantum extension specified by $\widehat{\mathcal{M}}_f$ has*

1. *reverse entropy $\widehat{\kappa}_f = \kappa_f$, where κ_f is the reverse entropy of the computation of f, derived in Supplementary Note 2, and*
2. *nonequilibrium cost $\widehat{c}_f(F) = \kappa_f + \log F$.*

The second equality holds for every F in the interval $[F_{\min}, F_{\max}]$, with $F_{\min} = 2^{c_{\min}-\kappa_f}$ and $F_{\max} = 1$.

Proof. Since the set of input states for the task $\rho_x \mapsto \mathcal{M}_f(\rho_x)$ includes the classical states $(|x\rangle\langle x|)_{x=1}^{d_A}$, the reverse entropy of the quantum-extended task, denoted by $\kappa_{\widehat{\mathcal{M}}_f}$, is generally larger than the reverse entropy of the original classical computation task, namely

$$\widehat{\kappa}_f \geq \kappa_f \,. \tag{60}$$

Evaluating the bound (25) at $F = 1$, we then obtain the inequality

$$\widehat{c}_f(1) \geq \widehat{\kappa}_f \geq \kappa_f \,. \tag{61}$$

On the other hand, the nonequilibrium cost of the channel $\widehat{\mathcal{M}}_f$ is exactly the same as the nonequilibrium cost of the channel $\mathcal{M}_f$ in Eq. (53), because the two channels act in the same way on the Gibbs state. Hence, we have the equality

$$c(\widehat{\mathcal{M}}_f, \Pi_A) = c(\mathcal{M}_f, \Pi_A) = \kappa_f \,, \tag{62}$$

where the second equality follows from Eq. (56).

Since the channel $\widehat{\mathcal{M}}_f$ achieves the state transformation task $\rho_x \mapsto \widehat{\mathcal{M}}_f(\rho_x)$ with unit accuracy, the above equality implies the bound

$$\widehat{c}_f(1) \leq c(\widehat{\mathcal{M}}_f, \Pi_A) = \kappa_f \,. \tag{63}$$

Comparing Eqs. (61) and (63), we obtain the following chain of inequalities

$$\kappa_f \leq \widehat{\kappa}_f \leq \widehat{c}_f(1) \leq c(\widehat{\mathcal{M}}_f, \Pi_A) = \kappa_f \,, \tag{64}$$

which imply the equalities

$$\widehat{\kappa}_f = \kappa_f \tag{65}$$

and

$$\widehat{c}_f(1) = \widehat{\kappa}_f \,. \tag{66}$$

The second equality implies that the bound (25) holds at $F = 1$ with the equality sign. Hence, Theorem 1 guarantees that the bound (25) holds for every F in the interval $[F_{\min}, 1]$, and $\widehat{c}_f(F) = \widehat{\kappa}_f + \log F \equiv \kappa_f + \log F$. □

Erasure of quantum states. Here we show that the work cost for the approximate erasure of a d-dimensional quantum system to the ground state is

$$W_{\text{erase}}(F) = \frac{\Delta A}{kT} + \ln F \,. \tag{67}$$

where ΔA is the difference between the free energy of the ground state and the free energy of the Gibbs state.

The erasure task corresponds to the state transformation $\rho \mapsto |0\rangle\langle 0|$, where ρ is an arbitrary pure state. As the figure of merit for approximate erasure, we consider the erasure fidelity $F_{\text{erase}}(\mathcal{M}) = \min_\rho \langle 0|\, \mathcal{M}(\rho)\, |0\rangle$, where $\mathcal{M}$ is the quantum channel used to implement the erasure task.

Lemma 3. *The reverse entropy of the erasure task is* $\kappa_{\rm erase} = \frac{\Delta A}{kT\,\ln 2}$.

Proof. The state transformation $\rho \mapsto |0\rangle\langle 0|$ is the quantum extension of the classical deterministic computation $f_0 : x \mapsto f(x) = 0$, where the classical input $x \in \{0, \dots, d-1\}$ is encoded in the computational basis state $|x\rangle$, as discussed in the previous section of this Supplementary Note. Hence, the reverse entropy of the erasure task can be computed with Eqs. (65) and (32), which give

$$\begin{aligned} \kappa_{\rm erase} &= D_{\max}(p_{f_0} \| g_B) \\ &= \log \frac{1}{g_B(0)} \\ &= \log[e^{E_0/(kT)}\, Z] \\ &= \frac{E_0}{kT}\, \log e + \log Z \\ &= \frac{A_0 - A_{\rm Gibbs}}{kT\,\ln 2} \\ &= \frac{\Delta A}{kT\,\ln 2}\,, \end{aligned} \tag{68}$$

where A_0 (respectively, $A_{\rm Gibbs}$) is the free energy of the state $|0\rangle\langle 0|$ (respectively, Γ), the free energy of a generic state ρ being defined as $A(\rho) := E(\rho) - kT\, S(\rho)$ with $E(\rho) = \operatorname{Tr}[H\,\rho]$ and $S(\rho) = -\operatorname{Tr}[\rho \ln \rho]$. □

Lemma 4. *The nonequilibrium cost and the work cost of erasure are*

$$c_{\rm erase}(F) = \kappa_{\rm erase} + \log F \qquad \text{and} \qquad W_{\rm erase}(F) = kT \ln 2\, c_{\rm erase}(F)\,, \tag{69}$$

respectively These expressions hold for every F *in the interval* $[F_{\min}, F_{\max}]$, *with* $F_{\min} = 2^{-\kappa_{\rm erase}} = e^{-\Delta A/(kT)}$ *and* $F_{\max} = 1$.

Proof. By Proposition 4, every quantum extension of a classical deterministic computation has cost $c_f(F) = \kappa_f + \log F$. In the special case of erasure, the minimum nonequilibrium cost is

$$c_{\rm erase}(F) = \kappa_{\rm erase} + \log F\,. \tag{70}$$

The quality holds for every F in the interval $[F_{\min}, F_{\max}]$, with $F_{\max} = 1$ and $F_{\min} = 2^{c_{\min} - \kappa_{\rm erase}} = 2^{-\kappa_{\rm erase}} = e^{-\Delta A/(kT)}$ (having used Lemma 2) for the expression of $F_{\min}$, plus the fact that the input subspace for the erasure task is the whole Hilbert space, and therefore the minimum nonequilibrium cost is $c_{\min} = 0$ by Lemma 1).

We now prove the equality $W_{\rm erase}(F) = kT \ln 2\, c_{\rm erase}(F)$. To this purpose, we consider the one-parameter family of quantum channels $\mathcal{M}_F$ defined in Eq. (40). In the specific case of erasure, the expression of the channel $\mathcal{M}_F$ is

$$\mathcal{M}_F(\rho) = \rho_F \quad \forall \rho \tag{71}$$

with

$$\rho_F := F\,|0\rangle\langle 0| + (1-F)\,\chi \qquad \text{and} \qquad \chi := \frac{\Gamma - F_{\min}\,|0\rangle\langle 0|}{1 - F_{\min}}\,. \tag{72}$$

(see Lemma 2 for the proof that χ is a valid quantum state).

The proof of Theorem 1 shows that the channel $\mathcal{M}_F$ has fidelity at least F and satisfies the equality

$$c(\mathcal{M}_F, \Pi_A) = c_{\rm erase}(F)\,. \tag{73}$$

On the other hand, Eq. (71) implies that the nonequilibrium cost of the channel $\mathcal{M}_F$ is

$$c(\mathcal{M}_F, \Pi_A) = D_{\max}(\mathcal{M}_F(\Gamma)\,\|\,\Gamma) = D_{\max}(\rho_F \| \Gamma)\,. \tag{74}$$

One way to realise the channel $\mathcal{M}_F$ in Eq. (71) is to prepare the state ρ_F and to swap it with the state of the input. The work cost of this realisation is equal to the work cost of generating the state ρ_F, which is given by $kT\,\ln 2\, D_{\max}(\rho_F \| \Gamma)$ [4]. Since $W_{\rm erase}(F)$ is the minimum work cost of erasure, we have the bound

$$W_{\rm erase}(F) \le kT\,\ln 2\, D_{\max}(\rho_F \| \Gamma) = kT\,\ln 2\, c(\mathcal{M}_F, \Pi_A) = kT\,\ln 2\, c_{\rm erase}(F)\,. \tag{75}$$

On the other hand, the nonequilibrium cost times $kT \ln 2$ is a lower bound to the work cost. Hence, we obtained the equality $W_{\rm erase}(F) = kT\,\ln 2\, c_{\rm erase}(F)$. □

Combining Lemmas 3 and 4 we finally obtain Eq. (67).

SUPPLEMENTARY NOTE 5: THE NONEQUILIBRIUM COST OF QUANTUM CLONING

Here we establish that *(i)* the reverse entropy of quantum cloning is equal to the reverse entropy of classical cloning, and *(ii)* the bound (4) in the main text is attainable for quantum cloning.

Our strategy is to evaluate the nonequilibrium cost of the optimal quantum cloner [5], and to infer from it the value of the reverse entropy, and the attainability of the bound (4) in the main text.

The nonequilibrium cost of the optimal cloner

Here we calculate the nonequilibrium cost of the optimal universal quantum cloning machine $\mathcal{M}_{\rm opt}$ by Werner [5], which transforms N copies of an arbitrary pure state ψ_x to $N' \geq N$ approximate copies of the same state. The cloner is described by the following quantum channel,

$$\mathcal{M}_{\rm opt}(\psi_x^{\otimes N}) = \frac{d_N}{d_{N'}} P_{N'} \left(\psi_x^{\otimes N} \otimes I^{\otimes N'-N}\right) P_{N'}\,, \tag{76}$$

where P_k is the projector onto the totally symmetric subspace of the k tensor product of k systems, and $d_k := \mathrm{Tr}[P_k]$ is the dimension of the totally symmetric subspace.

Here we consider non-interacting d-level systems, each with the same individual Hamiltonian $H = \sum_{i=0}^{d-1} E_i \,|i\rangle\langle i|$. We label the eigenvalues in increasing order, with $E_0 \leq E_1 \leq \cdots \leq E_{d-1} =: E_{\max}$.

In the following, we will denote by $\Gamma := e^{-\beta H}/\,\mathrm{Tr}\big[e^{-\beta H}\big]$ $(Z := \mathrm{Tr}\big[e^{-\beta H}\big])$ the single-particle Gibbs state (partition function), by $E_{\rm Gibbs} := \mathrm{Tr}[H\Gamma]$, $S_{\rm Gibbs} = -\,\mathrm{Tr}[\Gamma \ln \Gamma]$, and $A_{\rm Gibbs} := E_{\rm Gibbs} - kT\, S_{\rm Gibbs} = -kT \ln Z$, the Gibbs state energy, entropy, and free energy, respectively.

Theorem 2 (nonequilibrium cost of the optimal universal cloner)**.** *The nonequilibrium cost of the optimal universal cloner $\mathcal{M}_{\rm opt}$ in Eq. (76) is*

$$c(\mathcal{M}_{\rm opt}, \Pi_A) = \frac{\Delta N\, \Delta A_{\max}}{kT \ln 2} + \log F_{\max}\,, \tag{77}$$

where $F_{\max} := \log \frac{d_N}{d_{N'}}$ is the optimal cloning fidelity, $\Delta N := N' - N$ is the number of extra copies, and $\Delta A_{\max} := A_{\max} - A_{\rm Gibbs}$ is the difference between the maximum free energy of a single-copy state, given by $A_{\max} := E_{\max}$, and the free energy of the single-copy Gibbs state, given by $A_{\rm Gibbs} := -kT \ln Z$.

Proof. For the universal cloner, the input subspace is the totally symmetric subspace, and therefore $\Pi_A = P_N$. Hence, the nonequilibrium cost (6) reads

$$c(\mathcal{M}_{\rm opt}, P_N) = \log \left\| \left(\Gamma^{\otimes N'}\right)^{-1} \mathcal{M}_{\rm opt}\left(P_N\, \Gamma^{\otimes N}\, P_N\right) \left(\Gamma^{\otimes N'}\right)^{-1} \right\|\,. \tag{78}$$

The projected Gibbs state $P_N\, \Gamma^{\otimes N}\, P_N$ can be written as

$$P_N\, \Gamma^{\otimes N}\, P_N = \frac{1}{Z^N} \sum_{\vec{n} \in \mathsf{S}_{N,d}} e^{-\beta \vec{n}\cdot\vec{E}}\; |N, \vec{n}\rangle\langle N, \vec{n}|\,, \tag{79}$$

where $|N,\vec{n}\rangle$ denotes the normalised symmetric state with occupation number n_j in the j-th mode, $\vec{n} := (n_0, \dots, n_{d-1})$ a partition of N into d nonnegative integers, $\mathsf{S}_{N,d}$ is the set of all such partitions, and $\vec{E} = (E_0, \dots, E_{d-1})$ is the vector of single-system energy eigenstates.

The quantum cloner transforms the input state $|N,\vec{n}\rangle$ into

$$\begin{aligned}\mathcal{M}_{\rm opt}(\,|N,\vec{n}\rangle\langle N,\vec{n}|) &= \frac{d_N}{d_{N'}} P_{N'} \left(|N,\vec{n}\rangle\langle N,\vec{n}| \otimes I^{\otimes \Delta N}\right) P_{N'} \\ &= \frac{d_N}{d_{N'}} \sum_{\vec{k},\vec{l} \in \mathsf{S}_{N',d}} |N',\vec{k}\rangle\langle N',\vec{l}| \quad \langle N',\vec{k}| \left(|N,\vec{n}\rangle\langle N,\vec{n}| \otimes I^{\otimes \Delta N}\right) |N',\vec{l}\rangle\,, \end{aligned} \tag{80}$$

with

$$\begin{aligned}\langle N',\vec{k}|\,(|N,\vec{n}\rangle\langle N,\vec{n}|\otimes I^{\otimes\Delta N})\,|N',\vec{l}\rangle &= \sum_{\vec{r}\in\mathsf{S}_{\Delta N,d}} \langle N',\vec{k}|\,\big(\,|N,\vec{n}\rangle\langle N,\vec{n}|\otimes|\Delta N,\vec{r}\rangle\langle\Delta N,\vec{r}\,|\,\big)\,|N',\vec{l}\rangle\\ &=\delta_{\vec{k},\vec{l}}\,\left|\Big(\langle N,\vec{n}|\otimes\langle\Delta N,\vec{k}-\vec{n}|\Big)\,|N',\vec{k}\rangle\right|^2\\ &=\delta_{\vec{k},\vec{l}}\,\frac{\binom{\vec{k}}{\vec{n}}}{\binom{N'}{N}}\qquad \binom{\vec{k}}{\vec{n}}:=\prod_{j=0}^{d-1}\binom{k_j}{n_j}\,. \end{aligned} \tag{81}$$

Hence, we have

$$\mathcal{M}_{\rm opt}\left(P_N\,\Gamma^{\otimes N}\,P_N\right)=\frac{1}{Z^N}\frac{d_N}{d_{N'}}\sum_{\vec{n}\in\mathsf{S}_{N,d}}\sum_{\vec{n}'\in S_{N',d}} e^{-\beta\vec{n}\cdot\vec{E}}\,\frac{\binom{\vec{n}'}{\vec{n}}}{\binom{N'}{N}}\,|N',\vec{n}'\rangle\,\langle N',\vec{n}'|\,, \tag{82}$$

and

$$\left(\Gamma^{\otimes N'}\right)^{-\frac12}\mathcal{M}_{\rm opt}\left(P_N\,\Gamma^{\otimes N}\,P_N\right)\left(\Gamma^{\otimes N'}\right)^{-\frac12}=Z^{\Delta N}\,\frac{d_N}{d_{N'}}\sum_{\vec{n}'\in S_{N',d}}\left(\sum_{\vec{n}\in\mathsf{S}_{N,d}} e^{\beta\,(\vec{n}'-\vec{n})\cdot\vec{E}}\,\frac{\binom{\vec{n}'}{\vec{n}}}{\binom{N'}{N}}\right)|N',\vec{n}'\rangle\,\langle N',\vec{n}'|\,. \tag{83}$$

The norm of the above operator is given by

$$\left\|\left(\Gamma^{\otimes N'}\right)^{-\frac12}\mathcal{M}_{\rm opt}\left(P_N\,\Gamma^{\otimes N}\,P_N\right)\left(\Gamma^{\otimes N'}\right)^{-\frac12}\right\|=Z^{\Delta N}\,\frac{d_N}{d_{N'}}\max_{\vec{n}'\in S_{N',d}}\left(\sum_{\vec{n}\in\mathsf{S}_{N,d}} e^{\beta\,(\vec{n}'-\vec{n})\cdot\vec{E}}\,\frac{\binom{\vec{n}'}{\vec{n}}}{\binom{N'}{N}}\right).$$

Note that the binomial coefficient in the numerator of the r.h.s. is nonzero only if all the entries of the vector $\vec{n}'-\vec{n}$ are nonnegative. Under this condition, we have the inequality $e^{\beta(\vec{n}'-\vec{n})\,\vec{E}}\le e^{\beta\Delta N\,E_{\max}}$, and therefore the norm is upper bounded as

$$\begin{aligned}\left\|\left(\Gamma^{\otimes N'}\right)^{-\frac12}\mathcal{M}_{\rm opt}\left(P_N\,\Gamma^{\otimes N}\,P_N\right)\left(\Gamma^{\otimes N'}\right)^{-\frac12}\right\| &\le Z^{\Delta N}\,\frac{d_N}{d_{N'}}\,e^{\beta\,\Delta N\,E_{\max}}\max_{\vec{n}'\in S_{N',d}}\left(\sum_{\vec{n}\in\mathsf{S}_{N,d}}\frac{\binom{\vec{n}'}{\vec{n}}}{\binom{N'}{N}}\right)\\ &=Z^{\Delta N}\,\frac{d_N}{d_{N'}}\,e^{\beta\,\Delta N\,E_{\max}}\,,\end{aligned} \tag{84}$$

where the last equality follows from the Chu-Vandermonde identity. The upper bound is attained by choosing the vector $\vec{n}'$ with $n'_{d-1}=N'$ and $n_j=0\,,\forall j\neq d-1$.

Summarising, we obtained the equality

$$\begin{aligned}c(\mathcal{M}_{\rm opt},P_N)&=\Delta N\,\log Z+\log\frac{d_N}{d_{N'}}+\beta\,\Delta N\,E_{\max}\,\log e\\ &=\beta\,\log e\,\Delta N\,(E_{\max}-A_{\rm Gibbs})+\log F_{\max}\\ &\equiv\frac{\Delta N\,\Delta A_{\max}}{kT\,\ln 2}+\log F_{\max}\,.\end{aligned} \tag{85}$$

□

The reverse entropy of quantum cloning

Using the result of the previous subsection, we now show that the reverse entropy of quantum cloning coincides with the reverse entropy of classical cloning. From the main text, we have the bound

$$\kappa_{\rm clon}^{\rm Q} \geq \kappa_{\rm clon}^{\rm C} = \frac{\Delta N\,\Delta A_{\rm max}}{kT\,\ln 2}\,. \tag{86}$$

On the other hand, applying the bound (25) to the optimal cloning channel $\mathcal{M}_{\rm opt}$ yields the inequality

$$c(\mathcal{M}_{\rm opt},\Pi_A) \geq c_{\rm clon}(F_{\rm max}) \geq \kappa_{\rm clon}^{\rm Q} + \log F_{\rm max}\,. \tag{87}$$

Substituting Eq. (77) into Eq. (87), we then obtain the bound

$$\kappa_{\rm clon}^{\rm Q} \leq \frac{\Delta N\,\Delta A_{\rm max}}{kT\,\ln 2} \equiv \kappa_{\rm clon}^{\rm C}\,. \tag{88}$$

Hence, we obtained the equality $\kappa_{\rm clon}^{\rm Q} = \kappa_{\rm clon}^{\rm C}$.

Achievability of the lower bound (25)

We conclude the section by showing that the bound (25) is attainable for every value of the fidelity in the interval $[F_{\rm min}, F_{\rm max}]$.

For the optimal cloning channel, the bound (25) reads $c(\mathcal{M}_{\rm opt},\Pi_A) \geq \frac{\Delta N\,\Delta A_{\rm max}}{kT\,\ln 2} + \log F_{\rm max}$. On the other hand, Eq. (77) shows that the bound is achieved with the equality sign. In other words, the bound (25) is attainable at $F = F_{\rm max}$. Using the attainability criterion provided in the main text, we can then conclude that the bound (25) is attainable for every value of F in the interval $[F_{\rm min}, F_{\rm max}]$.

SUPPLEMENTARY NOTE 6: CLONING WITH ENTANGLEMENT BINDING MACHINES

Bound on the reverse entropy of the transpose cloning task

Here we consider the task of transpose cloning, which consists in transforming N copies of a pure quantum state ρ_x into N' copies of its transpose state ρ_x^T. To estimate the reverse entropy of transpose cloning, we use the expression $\kappa_{{\rm clon}^*} = \max_{\mathbf{p}} H(A|B)_{\omega_{{\rm clon}^*,\mathbf{p}}}$ and we fix the prior probability distribution $\mathbf{p}$ to be the normalised Haar measure $p({\rm d}x)$. With this choice, the operator $\omega_{{\rm clon}^*,\mathbf{p}}$ is

$$\begin{aligned}\omega_{{\rm clon}^*,\mathbf{p}} &= \int p({\rm d}x)\ (\Gamma_A^{-1}\otimes\Gamma_B)^{1/2}\ \underbrace{\rho_x^T\otimes\cdots\otimes\rho_x^T}_{N+N'\ \text{times}}\,(\Gamma_A^{-1}\otimes\Gamma_B)^{1/2}\\ &= (\Gamma_A^{-1}\otimes\Gamma_B)^{1/2}\,\frac{P_{N+N'}}{d_{N+N'}}\,(\Gamma_A^{-1}\otimes\Gamma_B)^{1/2}\,,\end{aligned} \tag{89}$$

where $P_{N+N'}$ is the projector on the symmetric subspace of $N+N'$ systems, and $d_{N+N'} = {\rm Tr}[P_{N+N'}]$ is the dimension of the symmetric subspace.

Now, the condition $I_A\otimes\Lambda_B \geq \omega_{{\rm clon}^*,\mathbf{p}}$ is equivalent to

$$\Gamma_A\otimes\Gamma_B^{-1/2}\Lambda_B\Gamma_B^{-1/2} \geq \frac{P_{N+N'}}{d_{N+N'}}\,. \tag{90}$$

Recall that $\Gamma_A = \Gamma^{\otimes N}$, and notice that one has

$$\Gamma_A \geq g_{\rm min}^N\, I_A\,, \tag{91}$$

where $g_{\rm min} = e^{-\beta E_{\rm max}}/Z$ is the smallest probability in the Gibbs distribution. Then, the condition (90) is satisfied by the operator

$$\Lambda_B^{\rm min} := \frac{\Gamma_B^{1/2}P_{N'}\Gamma_B^{1/2}}{g_{\rm min}^N\, d_{N+N'}}\,, \tag{92}$$

where $P_{N'}$ is the projector on the symmetric subspace of N' systems.

Hence, the reverse entropy satisfies the condition

$$\begin{aligned}
\kappa_{\mathrm{clon}^*} &= -\min_{I_A\otimes\Lambda_B \geq \omega_{\mathrm{clon}*,\mathbf{p}}} \log \mathrm{Tr}[\Lambda_B] \\
&\geq -\log \mathrm{Tr}\left[\Lambda_B^{\min}\right] \\
&= \log \frac{g_{\min}^N\, d_{N+N'}}{\mathrm{Tr}[\Gamma_B P_{N'}]} \qquad (93)\\
&\geq \log \frac{g_{\min}^N\, d_{N+N'}}{g_{\max}^{N'}\, d_{N'}}\,, \qquad (94)
\end{aligned}$$

where $g_{\max} = e^{-\beta E_{\min}}/Z$ is the maximum probability in the Gibbs state, and $d_{N'} = \mathrm{Tr}[P_{N'}]$ is the dimension of the symmetric subspace of N' systems.

Rearranging the terms, we finally obtain the inequality

$$\begin{aligned}
\kappa_{\mathrm{clon}^*} &\geq \log g_{\min}^{N-N'} + \log \frac{d_{N+N'}\,(g_{\min}/g_{\max})^{N'}}{d_{N'}} \\
&= \kappa_{\mathrm{clon}} + \log \frac{d_{N+N'}\, e^{-\beta N' \Delta E}}{d_{N'}}\,, \qquad (95)
\end{aligned}$$

with $\Delta E := E_{\max} - E_{\min}$.

The above inequality implies that the nonequilibrium cost of the transpose cloning task is lower bounded as

$$c_{\mathrm{clon}^*}(F) \geq \kappa_{\mathrm{clon}} + \log F + \log \frac{d_{N+N'}\, e^{-\beta N'\, \Delta E}}{d_{N'}}\,. \qquad (96)$$

This bound applies to all entanglement binding machines for the task of quantum cloning, due to the general argument shown in the main text.

Achievability of the bounds (95) and (96) for fully degenerate systems

Here we show that the the bounds (95) and (96) are exactly achievable for fully degenerate systems, corresponding to $\Delta E = 0$.

Let $\mathcal{E}_{\mathrm{opt}}$ be the optimal state estimation channel [6], whose action on the symmetric subspace is defined by

$$\mathcal{E}_{\mathrm{opt}}(P_N \rho P_N) := d_N \int \mathrm{d}\psi\ \mathrm{Tr}\left[\psi^{\otimes N}\, P_N\rho P_N\right]\ \psi^{\otimes N'}\,, \qquad (97)$$

where $\psi := |\psi\rangle\langle\psi|$ denote the projector on a generic pure state $|\psi\rangle$, and $\mathrm{d}\psi$ is the normalised unitarily invariant measure on the set of pure states.

Then, define the channel $\mathcal{E}_{\mathrm{opt}^*}$ via the relation

$$\mathcal{E}_{\mathrm{opt}^*}(\rho) := [\mathcal{E}_{\mathrm{opt}}(\rho)]^T \qquad \forall \rho\,. \qquad (98)$$

This channel achieves the optimal fidelity $F_{\max} = d_N/d_{N+N'}$ for the transpose cloning task, which coincides with the optimal fidelity for implementing the original cloning task via state estimation [6].

For the channel $\mathcal{E}_{\mathrm{opt}^*}$, we have the bound

$$\begin{aligned}
c(\mathcal{E}_{\mathrm{opt}}, \Pi_A) &\geq c_{\mathrm{clon}^*}(F_{\max}) \geq \kappa_{\mathrm{clon}^*} + \log F_{\max} \geq (N'-N)\log d + \log \frac{d_{N+N'}}{d_{N'}} + \log \frac{d_N}{d_{N+N'}} \\
&= (N'-N)\log d + \log \frac{d_N}{d_{N'}}\,, \qquad (99)
\end{aligned}$$

where the second inequality follows from the bound (96) with $\Delta E = 0$.

On the other hand, the nonequilibrium cost of the channel $\mathcal{E}_{\rm opt^*}$ is

$$\begin{aligned} c(\mathcal{E}_{\rm opt^*}, \Pi_A) &= \log \left\| d^{N'-N}\, \mathcal{E}_{\rm opt^*}(P_N) \right\| \\ &= \log \left\| d^{N'-N}\, \frac{d_N}{d_{N'}} P_{N'} \right\| \\ &= (N'-N)\, \log d + \log \frac{d_N}{d_{N'}} \,. \end{aligned} \tag{100}$$

Combining Eqs. (99) and (100), we then obtain the equalities

$$c_{\rm clon^*}(F_{\rm max}) = \kappa_{\rm clon^*} + \log F_{\rm max} \tag{101}$$

and

$$\kappa_{\rm clon^*} = (N'-N) \log d + \log \frac{d_{N+N'}}{d_{N'}} \,. \tag{102}$$

In particular, Eq. (101) implies that the bound (25) for transpose cloning is attained at $F = F_{\rm max}$. Hence, Theorem 1 implies that the bound (25) for transpose cloning is attained for every value of the fidelity in the interval $[F_{\rm min}, F_{\rm max}]$, namely

$$c_{\rm clon^*}(F_{\rm max}) = \kappa_{\rm clon^*} + \log F_{\rm max} \tag{103}$$

Incidentally, we observe that the one-parameter family of quantum channels $\mathcal{M}_F$ defined in the proof of Theorem 1 consists of entanglement breaking channels. Hence, the optimal accuracy/nonequilibrium tradeoff for transpose cloning is achieved by an entanglement breaking (and, in particular, an entanglement binding) channel for every value of F.

SUPPLEMENTARY NOTE 7: STATE TRANSMISSION AND STATE TRANSPOSITION WITH ENTANGLEMENT BINDING MACHINES

Here we establish a bound on the nonequilibrium cost of entanglement binding (EB) machines in the state transmission task $\rho_x \mapsto \rho_x$, where ρ_x is an arbitrary pure state. The bound is derived from a bound on the nonequilibrium cost of arbitrary quantum machines for the state transposition task $\rho_x \mapsto \rho_x^T$. This task is a special case of the transpose cloning task considered in the Supplementary Note 6.

The logic of the derivation is as follows. As discussed in Methods, the minimum nonequilibrium cost of EB machines achieving fidelity F in a given task $\rho_x \mapsto \rho'_x$ coincides with the minimum nonequilibrium cost of EB machines achieving fidelity F in the transpose task $\rho_x \mapsto {\rho'_x}^T$. In turn, the minimum cost of EB machines is lower bounded by the minimum cost of arbitrary quantum machines, mathematically described by trace-preserving completely positive linear maps. Hence, we have the bound

$$c_{\rm transmit,\, eb}(F) \equiv c_{\rm transpose,\, eb}(F) \ge c_{\rm transpose}(F) \tag{104}$$

where $c_{\rm transmit,\, eb}(F)$ ($c_{\rm transpose,\, eb}(F)$) is the minimum nonequilibrium cost needed to achieve fidelity F for state transmission (state transposition) with EB machines, and $c_{\rm transpose}(F)$ is the minimum nonequilibrium cost needed to achieve fidelity F for state transposition with arbitrary quantum machines.

The main result of this section is the following bound

$$c_{\rm transpose}(F) \ge \log \left[\frac{(d^2F - d)^2}{4\gamma(1-F)} + F \right] \,, \tag{105}$$

with $\gamma := \left(\sum_{m=1}^{d-1} e^{-\frac{\Delta E_m}{2kT}} \right)^2$, $\Delta E_m := E_m - E_{m-1}$, and with the eigenvalues of the Hamiltonian ordered so that $E_0 \le E_1 \le \cdots \le E_{d-1}$. This bound implies that the nonequilibrium cost is strictly larger than zero whenever the fidelity satisfies the condition

$$F > \frac{d + 2\sqrt{\gamma}}{d^2 + 2\sqrt{\gamma}} =: F_* \,. \tag{106}$$

For qubits, we will show that the bound (105) is achievable for every Hamiltonian and for every value of the fidelity between $F_* = (1+\sqrt{\gamma})/(2+\sqrt{\gamma}) \equiv F_{\rm min}$ (the fidelity corresponding to the minimum nonequilibrium cost $c_{\rm min} = 0$) and $F_{\rm max} = 2/3$ (the maximum fidelity allowed by quantum mechanics, corresponding to the maximum nonequilibrium cost $c_{\rm max} = \log\big(\gamma^{-1} + 2)/3\big)$).

For $d = 2$, this bound coincides with Eq. (11) in the main text.

State transposition with bounded nonequilibrium resources

Our strategy to derive the bound (105) is to consider the maximisation of the transposition fidelity under a constraint on the nonequilibrium resources. We define the maximum fidelity as

$$F_{\rm transpose}(c) := \max_{\mathcal{M}\,:\,c(\mathcal{M},\Pi_A)\le c} \min_{\rho_x} {\rm Tr}\big[\rho_x^T\,\mathcal{M}(\rho_x)\big]\,, \tag{107}$$

where the minimisation runs over all pure states $\rho_x = |\psi_x\rangle\langle\psi_x|$, x being some parametrisation of the unit sphere. To derive a bound on $F_{\rm transpose}(c)$, we observe that the maximisation in Eq. (107) can be restricted without loss of generality to quantum channels $\mathcal{M}$ satisfying the covariance property $\mathcal{M}\circ\mathcal{U}_{\boldsymbol\theta} = \mathcal{U}_{\boldsymbol\theta}^T\circ\mathcal{M}$, where $\boldsymbol\theta = (\theta_0,\dots,\theta_{d-1}) \in [0,2\pi]^{\times d}$ is a vector of d phases, $\mathcal{U}_{\boldsymbol\theta}$ ($\overline{\mathcal{U}}_{\boldsymbol\theta}$) is the unitary channel defined by $\mathcal{U}_{\boldsymbol\theta}(\rho) := U_{\boldsymbol\theta}\rho U_{\boldsymbol\theta}^\dagger\,,\forall\rho$ ($\overline{\mathcal{U}}_{\boldsymbol\theta}(\rho) := \overline{U}_{\boldsymbol\theta}\rho\overline{U}_{\boldsymbol\theta}^T\,,\forall\rho$), and $U_{\boldsymbol\theta} := \sum_m e^{-i\theta_m}\,|m\rangle\langle m|$ ($\overline{U}_{\boldsymbol\theta} := \sum_m e^{i\theta_m}\,|m\rangle\langle m|$) (see e.g. [7]). The reduction to covariant channels can be made without loss of generality, because for every given channel $\mathcal{M}$, the covariant channel

$$\mathcal{M}' := \int_0^{2\pi}\frac{{\rm d}\theta_0}{2\pi}\cdots\int_0^{2\pi}\frac{{\rm d}\theta_{d-1}}{2\pi}\mathcal{U}_{\boldsymbol\theta}^T\circ\mathcal{M}\circ\mathcal{U}_{\boldsymbol\theta}\,, \tag{108}$$

satisfies the conditions $F_{\rm transpose}(\mathcal{M}') \ge F_{\rm transpose}(\mathcal{M})$ and $c(\mathcal{M}',\Pi_A) \le c(\mathcal{M},\Pi_A)$.

In the Choi representation, the covariance condition $\mathcal{M}\circ\mathcal{U}_{\boldsymbol\theta} = \mathcal{U}_{\boldsymbol\theta}^T\circ\mathcal{M}\,,\forall\boldsymbol\theta$ is equivalent to the commutation condition $[M, U_{\boldsymbol\theta}\otimes U_{\boldsymbol\theta}] = 0\,,\forall\boldsymbol\theta$, where M is the Choi operator of $\mathcal{M}$ (see e.g. [8]). Using this commutation, the Choi operators of covariant quantum channels (completely positive trace preserving maps) can be characterised as those with the following block diagonal form

$$\begin{aligned} M =& \sum_{m=0}^{d-1} p_{mm}\,|m\rangle\langle m|\otimes|m\rangle\langle m| \\ &+\sum_{m=1}^{d-1}\sum_{n:\,n<m}\Big(p_{mn}\,|m\rangle\langle m|\otimes|n\rangle\langle n| + p_{nm}\,|n\rangle\langle n|\otimes|m\rangle\langle m| + c_{mn}\,|m\rangle\langle n|\otimes|n\rangle\langle m| + \overline{c}_{mn}\,|n\rangle\langle m|\otimes|m\rangle\langle n|\Big)\,, \end{aligned} \tag{109}$$

where p_{mn} are probabilities satisfying the normalisation $\sum_m p_{mn} = 1\,\forall n$, and c_{mn} are complex coefficients satisfying the condition $|c_{mn}|^2 \le p_{mn}p_{nm}$.

For a generic state $|\psi\rangle = \sum_m \psi_m\,|m\rangle$, the fidelity of the covariant channel $\mathcal{M}$ satisfies the bound

$$\begin{aligned} F_\psi(\mathcal{M}) &:= {\rm Tr}\big[\,(|\psi\rangle\langle\psi|)^T\mathcal{M}(|\psi\rangle\langle\psi|)\big] \\ &= \langle\overline{\psi}|\langle\overline{\psi}|M|\overline{\psi}\rangle|\overline{\psi}\rangle \\ &= \sum_{m=0}^{d-1} p_{mm}\,|\psi_m|^4 + \sum_{m=1}^{d-1}\sum_{n<m}|\psi_m|^2|\psi_n|^2\left(p_{mn}+p_{nm}+c_{mn}+\overline{c}_{mn}\right) \qquad (110) \\ &\le \sum_{m=0}^{d-1} p_{mm}\,|\psi_m|^4 + \sum_{m=1}^{d-1}\sum_{n:\,n<m}|\psi_m|^2|\psi_n|^2\left(p_{mn}+p_{nm}+2\sqrt{p_{mn}p_{nm}}\right)\,, \qquad (111) \end{aligned}$$

which can be achieved with the appropriate choice of coefficients $c_{mn} = \sqrt{p_{mn}p_{nm}}$. In particular, a computational basis state $|m\rangle$ gives fidelity $F_m(\mathcal{M}) = p_{mm}$, while an equatorial state, with $|\psi_m| = 1/d\,\forall m$, gives fidelity

$$\begin{aligned} F_{\rm equatorial}(\mathcal{M}) &= \frac{\sum_{m=0}^{d-1}p_{mm} + \sum_m\sum_{n:\,n<m}p_{mn}+p_{nm}+2\sqrt{p_{mn}p_{nm}}}{d^2} \\ &= \frac{d+2\sum_{m=1}^{d-1}\sum_{n:\,n<m}\sqrt{p_{mn}p_{nm}}}{d^2}\,. \end{aligned} \tag{112}$$

The above fidelities, maximised over all channels with nonequilibrium cost bounded by c, provide upper bounds to the worst case fidelity $F_{\rm transpose}(c)$. To introduce the nonequilibrium constraint, we observe that

$$\Gamma^{-\frac12}\mathcal{M}(\Gamma)\Gamma^{-\frac12} = \Gamma^{-\frac12}\,{\rm Tr}_A[(I\otimes\Gamma^T)M]\Gamma^{-\frac12} = \sum_{m,n} p_{mn}\frac{g_n}{g_m}\,|m\rangle\langle m|\,, \tag{113}$$

where $g_m = e^{-E_m/(kT)}/Z$ are the eigenvalues of the Gibbs state, ordered so that $g_0 \leq g_1 \leq \cdots \leq g_{d-1}$. Hence, the nonequilibrium cost (6) is given by

$$c(\mathcal{M}, \Pi_A) = \max_m \log\left(\sum_n p_{mn} \frac{g_n}{g_m}\right) . \tag{114}$$

Note that, for every $m \geq 1$, one has the bounds

$$\sum_{n:n<m} p_{mn} \leq \frac{g_m}{g_{m-1}} \left(\sum_{n:n<m} p_{mn} \frac{g_n}{g_m}\right) \leq \frac{g_m}{g_{m-1}} \left(\sum_{n:n\neq m} p_{mn} \frac{g_n}{g_m}\right) \leq \frac{g_m}{g_{m-1}} \left(2^{c(\mathcal{M},\Pi_A)} - p_{mm}\right) \tag{115}$$

and

$$\sum_{n:n<m} \sqrt{p_{mn}p_{nm}} \leq \sqrt{\left(\sum_{n:n<m} p_{mn}\right)\left(\sum_{n:n<m} p_{nm}\right)} \leq \sqrt{\frac{g_m}{g_{m-1}} \left(2^{c(\mathcal{M},\Pi_A)} - p_{mm}\right)(1-p_{mm})} . \tag{116}$$

Later we will see that this bound is achievable for qubits (but generally not for higher dimensional systems). Using this bound, the equatorial fidelity is upper bounded as

$$F_{\text{equatorial}}(\mathcal{M}) \leq \frac{d + 2\sum_{m=1}^{d-1} \sqrt{\frac{g_m}{g_{m-1}} \left(2^{c(\mathcal{M},\Pi_A)} - p_{mm}\right)(1-p_{mm})}}{d^2} \tag{117}$$

Now, suppose that a channel $\mathcal{M}_0$ has nonequilibrium cost less than c, namely $c(\mathcal{M}_0, \Pi_A) \leq c$. Recall that p_{mm} is equal to the fidelity on the computational basis state $|m\rangle$, and therefore it is lower bounded by the worst case fidelity $F_{\text{transpose}}(c)$. Hence, we have the upper bound

$$\begin{aligned} F_{\text{transpose}}(c) \leq F_{\text{equatorial}}(\mathcal{M}_0) &\leq \frac{d + 2\sum_{m=1}^{d-1} \sqrt{\frac{g_m}{g_{m-1}} \left(2^c - F_{\text{transpose}}(c)\right)(1-F_{\text{transpose}}(c))}}{d^2} \\ &\leq \frac{d + 2\sqrt{\gamma \left(2^c - F_{\text{transpose}}(c)\right)(1-F_{\text{transpose}}(c))}}{d^2} , \end{aligned} \tag{118}$$

having defined $\gamma := \left(\sum_{m=1}^{d-1} \sqrt{g_m/g_{m-1}}\right)^2$. Solving the above inequality in $F_{\text{transpose}}(c)$, we obtain the bound

$$F_{\text{transpose}}(c) \leq \frac{d^3 - 2\gamma(1+2^c) + \sqrt{\left[d^3 - 2\gamma(1+2^c)\right]^2 - (d^4 - 4\gamma)(d^2 - 4\gamma 2^c)}}{d^4 - 4\gamma} , \tag{119}$$

valid for $F_c \leq 1/d$. Solving the inequality in c, instead, we obtain the bound

$$c \geq \log\left[F_{\text{transpose}}(c) + \frac{\left(d^2\, F_c - d\right)^2}{4\gamma(1-F_c)}\right] , \tag{120}$$

valid for $F_{\text{transpose}}(c) \leq 1/d$. This bound is equivalent to Eq. (105).

Achievability of the bound (105) for qubits

For $d = 2$, the bound (105) reads

$$c_{\text{transpose}}(F) \geq \log\left[F + e^{\frac{\Delta E}{kT}} \frac{(2F-1)^2}{(1-F)}\right] , \qquad \forall F \geq \frac{1}{2} . \tag{121}$$

We now show that the bound holds with the equality sign for all possible Hamiltonians, and for all values of F in the interval $[F_{\min}, F_{\max}]$, with

$$F_{\min} = \frac{e^{\frac{\Delta E}{kT}}+1}{2\,e^{\frac{\Delta E}{kT}}+1} \qquad \text{and} \qquad F_{\max} = \frac{2}{3}\,. \tag{122}$$

(cf. Eq. (106) and the discussion following it).

To prove the achievability of the bound, we consider the quantum channel $\mathcal{M}$ with Choi operator

$$\begin{aligned} M &= p_{00}\,|0\rangle\langle 0|\otimes|0\rangle\langle 0| + p_{11}\,|1\rangle\langle 1|\otimes|1\rangle\langle 1| \\ &\quad + p_{01}\,|0\rangle\langle 0|\otimes|1\rangle\langle 1| + p_{10}\,|1\rangle\langle 1|\otimes|0\rangle\langle 0| + \sqrt{p_{01}\,p_{10}}\,\Big(|0\rangle\langle 1|\otimes|1\rangle\langle 0| + |1\rangle\langle 0|\otimes|0\rangle\langle 1|\Big)\,, \end{aligned} \tag{123}$$

with

$$p_{11} = F\,, \qquad p_{01} = 1-F\,, \qquad p_{10} = \frac{(2F-1)^2}{1-F}\,, \qquad \text{and} \qquad p_{00} = \frac{3F-4F^2}{1-F}\,. \tag{124}$$

These parameters define a completely positive trace-preserving map whenever $p_{00} \ge 0$, that is, whenever $F \le 3/4$. In particular, they define a valid quantum channel whenever $F \le 2/3 \equiv F_{\max}$.

For the channel $\mathcal{M}$, the nonequilibrium cost, given by Eq. (114), is

$$\begin{aligned} c(\mathcal{M},\Pi_A) &= \max\left\{\log\left(p_{00}+p_{01}\frac{g_1}{g_0}\right),\, \log\left(p_{11}+p_{10}\frac{g_0}{g_1}\right)\right\} \\ &= \max\left\{\log\left[\frac{3F-4F^2}{1-F}+\frac{g_1}{g_0}\,(1-F)\right],\ \log\left[F+e^{\Delta E}\,\frac{(2F-1)^2}{1-F}\right]\right\} \end{aligned} \tag{125}$$

For every $F \ge \left(e^{\Delta E/(kT)}+1\right)/\left(2\,e^{\Delta E/(kT)}+1\right) \equiv F_{\min}$, one has the equality

$$c(\mathcal{M},\Pi_A) = \log\left[F+e^{\Delta E/(kT)}\,\frac{(2F-1)^2}{1-F}\right] \qquad \forall F \in [F_{\min}, F_{\max}]\,. \tag{126}$$

We now show that the worst case fidelity of the channel $\mathcal{M}$ is F. The fidelity of this channel on a generic state $|\psi\rangle = \psi_0\,|0\rangle + \psi_1\,|1\rangle$ is given by Eq. (110) and yields the relation

$$F_\psi(\mathcal{M}) = \sum_{m=0}^{1} p_{mm}\,|\psi_m|^4 + |\psi_0|^2|\psi_1|^2\left(\sqrt{p_{01}}+\sqrt{p_{10}}\right)^2\,, \tag{127}$$

Note that one has $\sqrt{p_{01}}+\sqrt{p_{10}} = (2F-1)/\sqrt{1-F}+\sqrt{1-F} = F/\sqrt{1-F}$, and therefore

$$F_\psi(\mathcal{M}) = F\,|\psi_1|^4 + \frac{3F-4F^2}{1-F}|\psi_0|^4 + |\psi_0|^2|\psi_1|^2\,\frac{F^2}{1-F}\,. \tag{128}$$

For every $F \le 2/3 \equiv F_{\max}$, the minimum of $F_\psi(\mathcal{M})$ is attained for $|\psi\rangle = |1\rangle$, whence one has

$$F_{\text{transpose}}(\mathcal{M}) = \min_{|\psi\rangle} F_\psi(\mathcal{M}) = F \qquad \forall F \le F_{\max}\,. \tag{129}$$

Summarising, Eqs. (126) and (129) imply that the bound (121) is achievable for every Hamiltonian and for every value of F in the interval $[F_{\min}, F_{\max}]$.

B. Bound on the nonequilibrium cost of state transmission/state transposition with entanglement binding channels

The bound (121), valid for arbitrary quantum channels, implies the bound

$$c_{\text{transmit,eb}}(F) \equiv c_{\text{transpose,eb}}(F) \ge \log\left[F+e^{\frac{\Delta E}{kT}}\,\frac{(2F-1)^2}{(1-F)}\right]\,, \tag{130}$$

on the nonequilibrium cost of every EB channel that transmits or transposes quantum states with fidelity F. We now show that this bound holds with the equality sign for every Hamiltonian and for every value of the fidelity between $F_{\min}$ and $F_{\max}$. Indeed, the channel $\mathcal{M}$ defined through Eq. (123) is entanglement binding for every $F \in [F_{\min}, F_{\max}]$: to check this, it is enough to evaluate the partial transpose of the Choi operator M on the output system, which is given by

$$\begin{aligned} M^{T_B} &= p_{00}\,|0\rangle\langle 0| \otimes |0\rangle\langle 0| + p_{11}\,|1\rangle\langle 1| \otimes |1\rangle\langle 1| \\ &\quad + p_{01}\,|0\rangle\langle 0| \otimes |1\rangle\langle 1| + p_{10}\,|1\rangle\langle 1| \otimes |0\rangle\langle 0| + \sqrt{p_{01}\,p_{10}}\,\Big(|0\rangle\langle 1| \otimes |0\rangle\langle 1| + |1\rangle\langle 0| \otimes |1\rangle\langle 0|\Big)\,. \end{aligned} \tag{131}$$

The Choi operator is positive if and only if $p_{01}\,p_{10} \le p_{00}\,p_{11}$, that is, if and only if $(2F-1)^2 \le \frac{3F^2-4F^3}{1-F}$. This inequality is satisfied in the interval $[(5-\sqrt{5})/10, (5+\sqrt{5})/10]$, which contains the interval $[F_{\min}, F_{\max}]$.

Summarising, the transposed Choi operator M^{T_B} is positive for every $F \in [F_{\min}, F_{\max}]$. Hence, the channel $\mathcal{M}$ is entanglement binding. Furthermore, we observe that, since M is a two-qubit operator, the Peres-Horodecki criterion [9, 10] implies that M is separable. In turn, separability of M implies that the channel $\mathcal{M}$ is entanglement-breaking [11], or equivalently, that $\mathcal{M}$ is a measure-and-prepare channel, of the form

$$\mathcal{M}(\rho) = \sum_{i=1}^{k} \mathrm{Tr}[P_i\,\rho]\,\sigma_i\,, \tag{132}$$

where $(P_i)_{i=1}^k$ are positive operators representing a quantum measurement, and $(\sigma_i)_{i=1}^k$ are quantum states. Operationally, this means that the channel $\mathcal{M}$ can be realised by performing a measurement on the input, and re-preparing the output in the state σ_i.

SUPPLEMENTARY NOTE 8: BOUND ON WORK EXTRACTION

Here we show how the result of Ref. [4] on work extraction can be retrieved from our main bound (25). To this purpose, it is useful to review the framework of Ref. [4], where the allowed operations on system S are obtained from a joint energy-preserving unitary evolution that couples system S with a heat bath B in the thermal state, and with a work register R, initially in an energy eigenstate. In this framework, obtaining work ΔW means transforming the state of the work register from an energy eigenstate with energy $W_{\rm out}$ to an energy eigenstate with energy $W_{\rm in}$, with $W_{\rm in} - W_{\rm out} = \Delta W$.

Crucially, the effective evolution from system S to the composite system SR, consisting of the system and the work register, is a quantum channel $\mathcal{N}$ satisfying the covariance property $\mathcal{N}(U_t \rho U_t^\dagger) = (U_t \otimes V_t)\,\mathcal{N}(\rho)\,(U_t \otimes V_t)^\dagger$ where $U_t = e^{-itH_S/\hbar}$ and $V_t = e^{-itH_R/\hbar}$ are the time evolution operators for the system and for the work register, respectively, and $t \in \mathbb{R}$ is an arbitrary evolution time.

Now, consider the task of extracting work from an initial state ρ. Since the channel $\mathcal{N}$ is covariant, the amount of work extracted from ρ is equal to the amount of work extracted from $\rho_t := U_t \rho U_t^\dagger$, and, in turn, is equal to the amount of work extracted from the average state

$$\langle \rho \rangle = \lim_{T\to\infty} \frac{1}{T} \int_{-T/2}^{T/2} \mathrm{d}t\, U_t \rho U_t^\dagger\,. \tag{133}$$

One of the results of Ref. [4] is that the maximum work extractable from ρ is given by the min relative entropy $D_{\min}(\langle\rho\rangle \| \Gamma) = \mathrm{Tr}[\Pi\,\Gamma]$, where Π is the projector on the support of $\langle\rho\rangle$. In the following, we will retrieve this result from our main bound (25), by constructing a suitable test.

Let $\mathcal{M}(\cdot) := \mathrm{Tr}_R[\mathcal{N}(\cdot)]$ be the quantum channel representing the effective evolution of the system in the transformation that extracts work ΔW. Without loss of generality, we can assume that every state σ with support contained in the support of $\langle\rho\rangle$ is mapped to the thermal state. Now, consider the test $\mathcal{T}$ consisting in applying channel $\mathcal{M}$ to the state $\sigma := \Pi\Gamma\Pi/\mathrm{Tr}[\Gamma\,\Pi]$, and then measuring an observable O. The exact choice of observable will turn out to be irrelevant.

The accuracy measure defined by this test is

$$\mathcal{F}_{\mathcal{T}}(\mathcal{M}) := \mathrm{Tr}[O\,\mathcal{M}(\sigma)] \equiv \mathrm{Tr}[O\,\Gamma]\,. \tag{134}$$

On the other hand, the reverse entropy of the task specified by the input state σ and by the observable O is

$$\begin{aligned}\kappa_{\mathcal{T}} &= -\log \mathrm{Tr}[O\,\Gamma] - D_{\max}(\sigma\|\Gamma)\\ &= -\log \mathrm{Tr}[O\,\Gamma] - \log \|\Gamma^{-1/2}\sigma\Gamma^{-1/2}\|\\ &= -\log \mathrm{Tr}[O\,\Gamma] - \log \left\|\frac{\Pi}{\mathrm{Tr}[\Pi\,\Gamma]}\right\|\\ &= -\log \mathrm{Tr}[O\,\Gamma] + \log \mathrm{Tr}[\Pi\,\Gamma]\\ &= -\log \mathrm{Tr}[O\,\Gamma] - D_{\min}(\langle\rho\rangle\|\Gamma)\,,\end{aligned} \tag{135}$$

where the first equality follows from Eq. (13) in the main text, the third equality follows from the fact that Π and Γ commute, and the last equality follows from the definition of $D_{\min}$. Hence, our main bound (25) becomes $c_{\mathcal{T}}(F) \geq \log F + \kappa_{\mathcal{T}} = -D_{\min}(\langle\rho\rangle\|\Gamma)$. Since $kT\ln 2\, c_{\mathcal{T}}(F)$ is a lower bound to the work cost, the work cost of channel $\mathcal{M}$ is upper bounded by $-kT\,\ln 2\, D_{\min}(\langle\rho\rangle\|\Gamma)$, meaning that at most work $kT\,\ln 2\, D_{\min}(\langle\rho\rangle\|\Gamma)$ can be extracted.

SUPPLEMENTARY NOTE 9: ERASURE WITH THE ASSISTANCE OF A QUANTUM MEMORY

Consider the task of erasing a quantum system S with the assistance of a quantum memory Q [12]. The task is to reset the state of system S to a fixed pure state η_S, while preserving the state of the memory Q, possibly including its correlations to an external reference system R. Mathematically, the task can be concisely described as: transform a given pure state $|\Psi\rangle_{SQR}$ into the state $\eta_S \otimes \rho_{QR}$, with $\rho_{QR} := \mathrm{Tr}_S[|\Psi\rangle\langle\Psi|_{SQR}]$, by operating only on the system S and on the memory Q. In the following, we will first discuss the exact case, and then consider its approximate version, making connection with the results of [12].

In the exact case, the erasure task is equivalent to implementing the state transformation $\rho_{x,SQ} \mapsto \eta_S \otimes \rho_{x,Q}$, $\rho_{x,Q} := \mathrm{Tr}_S[\rho_{x,SQ}]$, for every input state $\rho_{x,SQ}$ with support contained in the support of $\rho_{SQ} := \mathrm{Tr}_R[|\Psi\rangle\langle\Psi|_{SQR}]$. Let us denote by Π_{SQ} the projector on the support of ρ_{SQ}. Since we are interested in bounding the work cost, we will assume without loss of generality that the support of ρ_{SQ} is invariant under time translations, that is, $[\Pi_{SQ}, H_{SQ}] = 0$, where H_{SQ} is the joint Hamiltonian of the system and the memory. If this condition is not satisfied, the argument in Supplementary Note 7 shows that replacing the state ρ_{SQ} with its time average $\langle\rho_{SQ}\rangle$ does not affect the work cost.

Let $\mathcal{M}$ be any quantum channel that achieves perfect erasure for the states in the support of ρ_{SQ}. Proposition 1 in the main text then implies that the nonequilibrium cost of $\mathcal{M}$ satisfies the bound $c(\mathcal{M}, \Pi_{SQ}) \geq D_{\max}(\eta_S \otimes \rho_{x,Q}\|\Gamma_{SQ}) - D_{\max}(\rho_{x,SQ}\|\Gamma_{SQ})$ for every state $\rho_{x,SQ}$ with support in the given subspace. In particular, let us choose the input state

$$\widetilde{\Gamma}_{SQ} := \frac{\Pi_{SQ}\Gamma_{SQ}\Pi_{SQ}}{\mathrm{Tr}[\Pi_{SQ}\Gamma_{SQ}]}\,, \tag{136}$$

where Π_{SQ} is the projector on the subspace containing the possible input states. With this choice, the bound becomes $c(\mathcal{M}, \Pi_{SQ}) \geq D_{\max}(\eta_S \otimes \gamma_Q\|\Gamma_{SQ}) - D_{\max}(\widetilde{\Gamma}_{SQ}\|\Gamma_{SQ})$, with $\gamma_Q := \mathrm{Tr}_S[\widetilde{\Gamma}_{SQ}]$. In fact, explicit calculation from Eq. (6) shows that the inequality holds with the equality sign. Hence, the work cost for implementing the channel $\mathcal{M}$ upon the support of Π_{SQ}, denoted by $W(\mathcal{M}, \Pi_{SQ})$, satisfies the bound

$$\frac{W(\mathcal{M}, \Pi_{SQ})}{kT\ln 2} \geq D_{\max}(\eta_S \otimes \gamma_Q\|\Gamma_{SQ}) - D_{\max}(\widetilde{\Gamma}_{SQ}\|\Gamma_{SQ})\,. \tag{137}$$

When the Hamiltonian of system SQ is completely degenerate, the bound becomes

$$\begin{aligned}\frac{W(\mathcal{M}, \Pi_{SQ})}{kT\ln 2} &\geq \log\|\gamma_Q\| + \log \mathrm{Tr}[\Pi_{SQ}]\\ &= \log\|\mathrm{Tr}_S[\Pi_{SQ}]\|\\ &=: H_0(S|Q)_{\rho_{SQ}}\,,\end{aligned} \tag{138}$$

where $H_0(S|Q)_{\rho_{SQ}}$ is the conditional Rényi entropy of order $\alpha = 0$.

We now consider a relaxation of the erasure task where the input state may slightly differ from ρ_{SQ}, and the operation performed by the machine may slightly differ from the ideal erasure operation. To make connection with the results of [12], here we take the system to have fully degenerate Hamiltonian.

To define the relaxation, we consider an input state $\widehat{\rho}_{SQ}$ which deviates from ρ_{SQ} by at most ϵ with respect to the purified distance [13], defined as $P(\rho, \widehat{\rho}) := \sqrt{1 - F(\rho, \widehat{\rho})}$, where $F(\rho, \widehat{\rho}) := \left(\|\sqrt{\rho}\sqrt{\widehat{\rho}}\|_1 + \sqrt{(1-\mathrm{Tr}[\rho])(1-\mathrm{Tr}[\widehat{\rho}])}\right)^2$

is the (generalised) fidelity and $\|O\|_1 := \mathrm{Tr}\Big[\sqrt{O^\dagger O}\Big]$ is the trace norm. Moreover, we consider a quantum channel $\widehat{\mathcal{M}}$ that implements an approximate erasure on a purification $\widehat{\Psi}_{SQR}$ of the state $\widehat{\rho}_{SQ}$, that is

$$T((\widehat{\mathcal{M}} \otimes \mathcal{I}_R)(\widehat{\Psi}_{SQR}), \eta_S \otimes \mathrm{Tr}_S[\widehat{\Psi}_{SQR}]) \leq \delta\,, \tag{139}$$

where $T(\rho,\sigma) := \|\rho - \sigma\|_1/2$ is the trace distance.

For the task of implementing the approximate erasure $\widehat{\mathcal{M}}$ on the approximate input state $\widehat{\rho}_{SQ}$, Proposition 1 in the main text implies that the nonequilibrium cost for fully degenerate Hamiltonians is lower bounded as $c(\widehat{\mathcal{M}}, \widehat{\Pi}_{SQ}) \geq D_{\max}(\widehat{\mathcal{M}}(\widehat{\pi}_{SQ})\|I_{SQ}/d_{SQ}) - D_{\max}(\widehat{\pi}_{SQ}\|I_{SQ}/d_{SQ})$, where d_{SQ} is the dimension of system SQ, and $\widehat{\pi}_{SQ} := \widehat{\Pi}_{SQ}/\,\mathrm{Tr}\Big[\widehat{\Pi}_{SQ}\Big]$ is the normalised quantum state proportional to the projector $\widehat{\Pi}_{SQ}$ on the support of $\widehat{\rho}_{SQ}$. Equivalently, the bound can be written as

$$c(\widehat{\mathcal{M}}, \widehat{\Pi}_{SQ}) \geq \Big\|\widehat{\mathcal{M}}(\widehat{\Pi}_{SQ})\Big\|\,. \tag{140}$$

Comparing the r.h.s. with Eq. (6) one can see that the bound holds with the equality sign.

The rest of our analysis follows an argument of Ref. [14], which is reproduced here for completeness in the scenario where two approximation parameters ϵ and δ are used. Let $\widehat{V} : \mathcal{H}_S \otimes \mathcal{H}_Q \to \mathcal{H}_S \otimes \mathcal{H}_Q \otimes \mathcal{H}_E$ be a Stinespring isometry for the channel $\widehat{\mathcal{M}}$, so that $\widehat{\mathcal{M}}(\widehat{\Pi}_{SQ}) = \mathrm{Tr}_E[\widehat{V}\widehat{\Pi}_{SQ}\widehat{V}^\dagger]$. Here we choose the environment to have dimension larger than the dimension of system S, so that the environment E can also be used to implement the ideal erasure operation by embedding the state of system S into E.

In terms of the Stinespring isometry, the bound (140) becomes

$$\begin{aligned} c(\widehat{\mathcal{M}}, \widehat{\Pi}_{SQ}) &\geq \Big\|\mathrm{Tr}_E[\widehat{V}\widehat{\Pi}_{SQ}\widehat{V}^\dagger]\Big\| \\ &\equiv H_0(E|SQ)_{\widehat{V}\widehat{\rho}_{SQ}\widehat{V}^\dagger}\,. \end{aligned} \tag{141}$$

Since the Rényi entropy of order 0 is at least as large as the Rényi entropy of order 1/2, one also has

$$c(\widehat{\mathcal{M}}, \widehat{\Pi}_{SQ}) \geq H_{1/2}(E|SQ)_{\widehat{V}\widehat{\rho}_{SQ}\widehat{V}^\dagger}\,. \tag{142}$$

Now, let $\widehat{\Psi}_{EQR}$ be the state obtained from $\widehat{\Psi}_{SQR}$ by embedding system S into the environment E. Uhlmann's theorem implies that the isometry V can be chosen in such a way that the fidelity between the pure state $(\widehat{V} \otimes I_R)\widehat{\Psi}_{SQR}(\widehat{V}^\dagger \otimes I_R)$ and the pure state $\eta_S \otimes \widehat{\Psi}_{EQR}$ is equal to the fidelity between their marginal states $(\widehat{\mathcal{M}} \otimes \mathcal{I}_R)(\widehat{\rho}_{SQR})$ and $\eta_S \otimes \widehat{\rho}_{QR}$, respectively. Then, Eq. (139) and the Fuchs-van de Graaf inequality implies that the fidelity is at least $(1-\delta)^2$. Hence, the Fuchs-van de Graaf inequality implies that the purified distance between the states $(\widehat{V} \otimes I_R)\widehat{\Psi}_{SQR}(\widehat{V}^\dagger \otimes I_R)$ and $\eta_S \otimes \widehat{\Psi}_{EQR}$ is at most $\sqrt{2\delta}$. Since the purified distance is nonincreasing under partial trace, one also has $P(\widehat{V}\widehat{\rho}_{SQ}\widehat{V}^\dagger, \eta_S \otimes \widehat{\rho}_{QE}) \leq \sqrt{2\delta}$, where $\widehat{\rho}_{QE}$ is the state obtained from $\widehat{\rho}_{SQ}$ by embedding system E into the environment.

The triangle inequality for the purified distance yields the bound $P(\widehat{V}\widehat{\rho}_{SQ}\widehat{V}^\dagger, \eta_S \otimes \rho_{QE}) \leq P(\widehat{V}\widehat{\rho}_{SQ}\widehat{V}^\dagger, \eta_S \otimes \widehat{\rho}_{QE}) + P(\eta_S \otimes \widehat{\rho}_{QE} \otimes \eta_S \otimes \rho_{QE}) \leq \sqrt{2\delta} + \epsilon$. Using this fact, the bound Eq. (142) can be relaxed to

$$c(\widehat{\mathcal{M}}, \widehat{\Pi}_{SQ}) \geq \min_{\rho_{SQE}\colon P(\rho_{SQE}, \eta_S \otimes \rho_{QE}) \leq \sqrt{2\delta}+\epsilon} H_{1/2}(E|SQ)_{\rho_{SQE}} \tag{143}$$

$$\equiv H_{1/2}^{\sqrt{2\delta}+\epsilon}(E|SQ)_{\eta_S \otimes \rho_{QE}}\,, \tag{144}$$

where $H_{1/2}^{\sqrt{2\delta}+\epsilon}$ is the smooth conditional Rényi entropy of order 1/2. Note that the above expression can be further simplified, as one has the equality $H_{1/2}^{\sqrt{2\delta}+\epsilon}(E|SQ)_{\eta_S \otimes \rho_{QE}} = H_{1/2}^{\sqrt{2\delta}+\epsilon}(E|Q)_{\rho_{QE}} = H_{1/2}^{\sqrt{2\delta}+\epsilon}(S|Q)_{\rho_{SQ}}$ [14], which follows from the invariance under isometries of $H_{1/2}$ (cf. Section 5.3 of [15]). Since the nonequilibrium cost is a lower bound to the work cost, we obtained the relation $\frac{W(\widehat{\mathcal{M}}, \widehat{\Pi}_{SQ})}{kT \ln 2} \leq H_{1/2}^{\epsilon}(S|Q)_{\rho_{SQ}}$, which coincides with the upper bound from Ref. [12], up to logarithmic terms and to a slight redefinition of the approximation parameters.

SUPPLEMENTARY NOTE 10: STATE TRANSFORMATION TASKS VS INDIVIDUAL STATE TRANSITIONS

Here we discuss the relation between the nonequilibrium cost of a state transformation task $\rho_x \mapsto \rho'_x$, $\forall x \in \mathsf{X}$, and the nonequilibrium cost of the individual state transitions $\rho_x \mapsto \rho'_x$ corresponding to fixed values of x. In particular,

we show an example where the cost of each individual state transition is ≤ 0, while the cost of the overall state transformation task is strictly positive.

Relation between the reverse entropies

We start by proving the bound on the reverse entropy given in Eq. (19) of the Methods section:

Proposition 5. *Let $\mathcal{T}$ be an arbitrary state transformation task $\rho_x \mapsto \rho'_x$, $\forall x \in \mathsf{X}$. The reverse entropy $\kappa_{\mathcal{T}}$ satisfies the inequality*

$$\kappa_{\mathcal{T}} \geq \max_{x\in\mathsf{X}} -\log \mathrm{Tr}[O_x \Gamma_B] - D_{\max}(\rho_x \| \Gamma_A)\,, \tag{145}$$

where O_x is the observable used in the accuracy measure $\mathcal{F}_{\mathcal{T}}(\mathcal{M}) = \min_x \mathrm{Tr}[O_x \mathcal{M}(\rho_x)]$.

Proof. One has

$$\begin{aligned}\kappa_{\mathcal{T}} &= \max_{\mathbf{p}} H(A|B)_{\omega_{\mathcal{T},\mathbf{p}}}\\ &\geq H(A|B)_{\omega_{\mathcal{T},\mathbf{p}=(\delta_{x,x_0})}}\\ &= H(A|B)_{\Gamma_A^{-\frac{1}{2}}\rho_{x_0}^T\Gamma_A^{-\frac{1}{2}}\otimes\Gamma_B^{\frac{1}{2}}O_{x_0}\Gamma_B^{\frac{1}{2}}}\\ &= -\log\min\left\{\mathrm{Tr}[\Lambda_B]\mid (I_A\otimes\Lambda_B)\geq\Gamma_A^{-\frac{1}{2}}\rho_{x_0}^T\Gamma_A^{-\frac{1}{2}}\otimes\Gamma_B^{\frac{1}{2}}O_{x_0}\Gamma_B^{\frac{1}{2}}\right\}\,.\end{aligned}$$

To evaluate the minimum over Λ_B, one can take the trace on both sides of the constraint $(I_A \otimes \Lambda_B) \geq \Gamma_A^{-\frac{1}{2}}\rho_{x_0}^T\Gamma_A^{-\frac{1}{2}} \otimes \Gamma_B^{\frac{1}{2}}O_{x_0}\Gamma_B^{\frac{1}{2}}$, thus obtaining the operator inequality

$$\mathrm{Tr}[\Lambda_B]\, I_A \geq \mathrm{Tr}[O_{x_0}\Gamma_B]\,\Gamma_A^{-\frac{1}{2}}\rho_{x_0}^T\Gamma_A^{-\frac{1}{2}}\,, \tag{146}$$

which in turn implies

$$\mathrm{Tr}[\Lambda_B] \geq \mathrm{Tr}[O_{x_0}\Gamma_B]\,\left\|\Gamma_A^{-\frac{1}{2}}\rho_{x_0}^T\Gamma_A^{-\frac{1}{2}}\right\| = \mathrm{Tr}[O_{x_0}\Gamma_B]\,\left\|\Gamma_A^{-\frac{1}{2}}\rho_{x_0}\Gamma_A^{-\frac{1}{2}}\right\| = \mathrm{Tr}[O_{x_0}\Gamma_B]\,2^{D_{\max}(\rho_{x_0}\|\Gamma_A)}\,. \tag{147}$$

The bound is attained with the equality sign by setting $\Lambda_B = 2^{D_{\max}(\rho_{x_0}\|\Gamma_A)}\,\Gamma_B^{\frac{1}{2}}O_{x_0}\Gamma_B^{\frac{1}{2}}$. Hence, Eq. (146) becomes

$$\begin{aligned}\kappa_{\mathcal{T}} &\geq -\log\mathrm{Tr}[O_{x_0}\Gamma_B]\,2^{D_{\max}(\rho_{x_0}\|\Gamma_A)}\\ &= -\log\mathrm{Tr}[O_{x_0}\Gamma_B] - D_{\max}(\rho_{x_0}\|\Gamma_A) \qquad \forall x_0\in\mathsf{X}\,,\end{aligned} \tag{148}$$

which implies Eq. (145). □

In the Methods section of the main text, we have seen a choice of observables O_x for which Eq. (145) reduces to the entropic inequality

$$\kappa_{\mathcal{T}} \geq \max_{x\in\mathsf{X}} D_{\max}(\rho'_x\|\Gamma_B) - D_{\max}(\rho_x\|\Gamma_A)\,. \tag{149}$$

This inequality has an intuitive physical interpretation, relating the nonequilibrium cost of the task $\mathcal{T}$ to the deviation of the input and output states from the equilibrium state. However, it is important to stress that, in general, the deviation of the input/output states from the equilibrium state is not sufficient to evaluate the nonequilibrium cost of a given task. This fact is evident in the state transposition task $\rho_x \mapsto \rho_x^T$, where ρ_x is an arbitrary pure state: since $D_{\max}(\rho^T\|\Gamma) = D_{\max}(\rho\|\Gamma)$ for every quantum state ρ, Eq. (149) yields the trivial inequality $\kappa_{\mathrm{transpose}} \geq 0$, which is not tight, as we know that $\kappa_{\mathrm{transpose}} = \log[(d+1)/2]$ in the fully degenerate case (cf. the Results part in the main text).

The example of ideal transposition

For transposition, the nonequilibrium cost is nonnegative whenever the Hamiltonian is not fully degenerate (cf. Eq. (105)). Hence, the quantity $\max_x \Big[D_{\max}(\rho'_x\|\Gamma) - D_{\max}(\rho_x\|\Gamma)\Big]$ fails to detect the presence of a strictly positive nonequilibrium cost.

The same issue arises for other quantum versions of the relative entropy, including all the quantum Rényi relative entropies

$$D_\alpha(\rho\|\sigma) = \frac{\log \mathrm{Tr}\big[\rho^\alpha \sigma^{1-\alpha}\big]}{\alpha-1}\,, \qquad \alpha \ge 0, \alpha \ne 1\,, \tag{150}$$

as well as the "sandwiched" Rényi relative entropies [16, 17]

$$\widetilde{D}_\alpha(\rho\|\sigma) = \frac{\log \mathrm{Tr}\Big[(\sigma^{\frac{1-\alpha}{2\alpha}}\rho\sigma^{\frac{1-\alpha}{2\alpha}})^\alpha\Big]}{\alpha-1}\,, \qquad \alpha \ge 0, \alpha \ne 1\,, \tag{151}$$

which feature in the quantum second laws of Ref. [18] for $\alpha \ge 1/2$.

In these cases, one still has the equalities

$$D_\alpha(\rho^T\|\Gamma) = D_\alpha(\rho\|\Gamma) \qquad \text{and} \qquad \widetilde{D}_\alpha(\rho^T\|\Gamma) = \widetilde{D}_\alpha(\rho\|\Gamma) \quad \forall \alpha\,, \forall \rho\,, \tag{152}$$

valid whenever the transpose is defined in the eigenbasis of the Hamiltonian. Even more generally, the relation $\Delta(\rho^T\|\Gamma) = \Delta(\rho\|\Gamma)\,, \forall \rho$ holds for every function $\Delta(\rho\|\sigma)$ that is invariant under state space symmetries in the sense of Wigner's theorem (see e.g. [19]).

The example of approximate transposition

A natural question is whether the failure of the relative entropy to characterise the nonequilibrium cost of the transpose task is due to the fact that the ideal transposition $\rho_x \mapsto \rho_x^T$ is forbidden by quantum mechanics [20–22]. Instead of ideal transposition, one could consider some physical approximation of this impossible transposition task. The approximate transposition would then correspond to a valid quantum channel $\mathcal{M}$. The question is whether the change of relative entropy from the input to the output provides the correct value of the nonequilibrium cost. Here we show that, in general, the answer is negative.

To facilitate the evaluation of the relative entropies, we consider a restricted transposition task, where the input states are pure qubit states in the set $\mathsf{S} = \{|0\rangle\langle 0|\,, |1\rangle\langle 1|\} \cup \{|e_\theta\rangle\langle e_\theta|\}_{\theta\in[0,2\pi)}$, consisting of the computational basis states $|0\rangle$ and $|1\rangle$ and of the equatorial states $|e_\theta\rangle := (|0\rangle + e^{i\theta})/\sqrt{2}$. In this case, the nonequilibrium cost is still positive for every nondegenerate Hamiltonian: for $d = 2$, Eq. (105) indicates a positive value of the nonequilibrium cost for every value of the fidelity above $F_{\min}$. In particular, we will focus on the maximum value $F_{\max} = 2/3$, which is achieved by the channel $\mathcal{M}$ defined by [20–22]

$$\mathcal{M}(\rho) = \frac{I + \rho^T}{3} \qquad \forall \rho\,. \tag{153}$$

We will restrict our attention to the case where $\Gamma = \frac{2}{3}|0\rangle\langle 0| + \frac{1}{3}|1\rangle\langle 1|$, which considerably simplifies the calculations. In this case, one has

$$D_\alpha(\mathcal{M}(|0\rangle\langle 0|)\,\|\,\Gamma) - D_\alpha(|0\rangle\langle 0|\,\|\,\Gamma) = -D_\alpha(|0\rangle\langle 0|\,\|\,\Gamma) = \log\frac{2}{3} < 0 \tag{154}$$

$$D_\alpha(\mathcal{M}(|1\rangle\langle 1|)\,\|\,\Gamma) - D_\alpha(|1\rangle\langle 1|\,\|\,\Gamma) = \frac{\log[\left(\frac{2}{3}\right)^\alpha + \left(\frac{1}{3}\right)^\alpha\, 2^{1-\alpha}]}{\alpha-1} \le -H_\alpha\left(\frac{2}{3},\frac{1}{3}\right) < 0 \tag{155}$$

$$D_\alpha(\mathcal{M}(|e_\theta\rangle\langle e_\theta|)\,\|\,\Gamma) - D_\alpha(|e_\theta\rangle\langle e_\theta|\,\|\,\Gamma) = -H_\alpha\left(\frac{2}{3},\frac{1}{3}\right) < 0\,, \qquad \forall \theta \in [0,2\pi)\,, \tag{156}$$

where $H_\alpha\left(\frac{2}{3},\frac{1}{3}\right) := (1-\alpha)^{-1}\,\log\left[\left(\frac{2}{3}\right)^\alpha + \left(\frac{1}{3}\right)^\alpha\right]$ is the Rényi entropy of the binary probability distribution $(2/3, 1/3)$. Hence, the quantum Rényi relative entropies do not detect the positive work cost of the approximate transposition $\rho_x \mapsto \mathcal{M}(\rho_x)$ for all the states in S.

Let us now consider the sandwiched Rényi relative entropy $\widetilde{D}_\alpha$. For the states $|0\rangle$ and $|1\rangle$ the sandwiched Rényi relative entropies coincide with the quantum Rényi entropies. Hence, we only need to consider the case of the equatorial states $|e_\theta\rangle$. For $0 \le \alpha < 1$, we use the monotonicity of $\widetilde{D}_\alpha$ with respect to α [16] and the convergence to the von Neumann relative entropy $\lim_{\alpha\to1} \widetilde{D}_\alpha(\rho\|\sigma) = D(\rho\|\sigma) := \mathrm{Tr}[\rho\log\rho] - \mathrm{Tr}[\rho\log\sigma]\,\forall\rho,\sigma$ [16], which yield the bound

$$\widetilde{D}_\alpha(\mathcal{M}(|e_\theta\rangle\langle e_\theta|)\,\|\,\Gamma) \le D(\mathcal{M}(|e_\theta\rangle\langle e_\theta|)\,\|\,\Gamma) = \frac{1}{6}\,. \tag{157}$$

On the other hand, one has

$$\begin{aligned} -\widetilde{D}_\alpha(|e_\theta\rangle\langle e_\theta|\,\|\,\Gamma) &= \log\left[\frac{\left(\frac{2}{3}\right)^\beta + \left(\frac{1}{3}\right)^\beta}{2}\right]^{\frac{1}{\beta}} \qquad \beta := \frac{1-\alpha}{\alpha} \\ &\le \log\frac{2}{3}\,. \end{aligned} \tag{158}$$

Hence, we obtained the bound

$$\widetilde{D}_\alpha(\mathcal{M}(|e_\theta\rangle\langle e_\theta|)\,\|\,\Gamma) - \widetilde{D}_\alpha(|e_\theta\rangle\langle e_\theta|\,\|\,\Gamma) \le \frac{1}{6} + \log\frac{2}{3} \approx -0.418 < 0\,. \tag{159}$$

For $\alpha > 1$, we use the fact that the sandwiched divergence $\widetilde{D}_\alpha$ satisfies the data processing inequality $\widetilde{D}_\alpha(\mathcal{C}(\rho)\,\|\,\mathcal{C}(\sigma)) \le \widetilde{D}_\alpha(\rho\|\sigma)$ for every quantum channel $\mathcal{C}$ and for every pair of states ρ and σ [16, 17, 23, 24]. In particular, choosing $\mathcal{C}$ to be the partial dephasing channel $\mathcal{C}(\rho) := \frac{2}{3}\,\rho + \frac{1}{3}\sigma_z\rho\sigma_z$ (with $\sigma_z := |0\rangle\langle 0| - |1\rangle\langle 1|$), we obtain the bound

$$\widetilde{D}_\alpha(\mathcal{M}(|e_\theta\rangle\langle e_\theta|)\,\|\,\Gamma) = \widetilde{D}_\alpha(\mathcal{C}(|e_\theta\rangle\langle e_\theta|)\,\|\,\mathcal{C}(\Gamma)) \le \widetilde{D}_\alpha(|e_\theta\rangle\langle e_\theta|\,\|\,\Gamma)\,,$$

and therefore $\widetilde{D}_\alpha(\mathcal{M}(|e_\theta\rangle\langle e_\theta|)\,\|\,\Gamma) - \widetilde{D}_\alpha(|e_\theta\rangle\langle e_\theta|\,\|\,\Gamma) \le 0$.

Summarising, the change of sandwiched Rényi relative entropy from the input to the output is negative for every possible pair of states in S. Hence, the sandwiched Rényi relative entropies cannot detect the positive nonequilibrium cost of the approximate transposition $\rho_x \mapsto \mathcal{M}(\rho_x)$.

The above conclusions hold also if one considers the entropy of the Gibbs state relative to the input/output states, instead of the entropy of the input/output states relative to the Gibbs state. In this case, some care is required when dealing with the relative entropy $D_\alpha(\rho\||\psi\rangle\langle\psi|)$ for a pure state $|\psi\rangle$. Here we adopt the definition

$$D_\alpha(\rho\||\psi\rangle\langle\psi|) := \lim_{\epsilon\to0} D_\alpha\left(\rho\,\middle\|\,(1-\epsilon)\,|\psi\rangle\langle\psi| + \epsilon\,\frac{I - |\psi\rangle\langle\psi|}{d-1}\right) = \begin{cases} \frac{\log\langle\psi|\rho^\alpha|\psi\rangle}{\alpha-1} & 0 \le \alpha < 1 \\ \infty & \alpha > 1\,. \end{cases} \tag{160}$$

For the quantum Rényi relative entropies, one has the relations

$$D_\alpha(\Gamma\,\|\,\mathcal{M}(|0\rangle\langle 0|)) - D_\alpha(\Gamma\,\||0\rangle\langle 0|) = -D_\alpha(\Gamma\,\|\,|0\rangle\langle 0|) = \begin{cases} -\frac{\alpha}{1-\alpha}\log\frac{3}{2} & 0 \le \alpha < 1 \\ -\infty & \alpha > 1 \end{cases} \tag{161}$$

$$D_\alpha(\Gamma\,\|\,\mathcal{M}(|1\rangle\langle 1|)) - D_\alpha(\Gamma\,\||1\rangle\langle 1|) = \begin{cases} -\log\frac{3}{2} - \frac{1}{1-\alpha}\log\bigl(1 + 2^{2\alpha}\bigr) & 0 \le \alpha < 1 \\ -\infty & \alpha > 1 \end{cases} \tag{162}$$

$$D_\alpha(\Gamma\|\mathcal{M}(|e_\theta\rangle\langle e_\theta|)) - D_\alpha(\Gamma\||e_\theta\rangle\langle e_\theta|) = \begin{cases} -H_{1-\alpha}\left(\frac{3}{2}, \frac{1}{2}\right) < 0 & 0 \le \alpha < 1 \\ -\infty & \alpha > 1 \end{cases} \tag{163}$$

All these values are strictly negative.

The same conclusion applies to the sandwiched Rényi relative entropies, adopting the definition

$$\widetilde{D}_\alpha(\rho\||\psi\rangle\langle\psi|) := \lim_{\epsilon\to0} \widetilde{D}_\alpha\left(\rho\,\middle\|\,(1-\epsilon)\,|\psi\rangle\langle\psi| + \epsilon\,\frac{I - |\psi\rangle\langle\psi|}{d-1}\right) = \begin{cases} \frac{\alpha}{\alpha-1}\log\langle\psi|\rho|\psi\rangle & 0 \le \alpha < 1 \\ \infty & \alpha > 1\,. \end{cases} \tag{164}$$

(A more common definition is $\widetilde{D}_\alpha(\rho\||\psi\rangle\langle\psi|) = \infty$ whenever $\mathsf{Supp}(\rho) \not\subseteq \mathsf{Supp}(|\psi\rangle\langle\psi|)$. In this case, the difference $\widetilde{D}_\alpha(\Gamma\|\mathcal{M}(|e_\theta\rangle\langle e_\theta|)\,) - \widetilde{D}_\alpha(\Gamma\||e_\theta\rangle\langle e_\theta|\,)$ is trivially $-\infty$, and therefore cannot detect the positive nonequilibrium cost of the transposition task.)

We focus on the case of the equatorial states $|e_\theta\rangle$, because for the states $|0\rangle$ and $|1\rangle$ the sandwiched entropies coincide with the quantum Rényi relative entropies. For $0 \le \alpha < 1$, we use the equality

$$\widetilde{D}_\alpha(\Gamma \| |e_\theta\rangle\langle e_\theta|) = \frac{-\alpha}{\alpha - 1}\,, \tag{165}$$

and the inequality

$$\widetilde{D}_\alpha(\Gamma\|\Sigma\,) = \frac{1}{\alpha-1}\log \mathrm{Tr}\Big[\Big(\Sigma^{\frac{1-\alpha}{2\alpha}}\,\Gamma\,\Sigma^{\frac{1-\alpha}{2\alpha}}\Big)^\alpha\Big] \le \frac{1}{\alpha-1}\log \mathrm{Tr}\left[\left(\frac{1}{3}\,\Sigma^{\frac{1-\alpha}{\alpha}}\right)^\alpha\right] \qquad \forall \Sigma\,, \tag{166}$$

following from the operator monotonicity of the function $f(X) = X^\alpha$ and from the operator inequality $\Sigma^{\frac{1-\alpha}{2\alpha}}\,\Gamma\,\Sigma^{\frac{1-\alpha}{2\alpha}} \ge \frac{1}{3}\,\Sigma^{\frac{1-\alpha}{\alpha}}$, and from the fact that α is smaller than 1. Setting $\Sigma = \mathcal{M}(|e_\theta\rangle\langle e_\theta|)$ we obtain

$$\begin{aligned}
\widetilde{D}_\alpha(\Gamma\|\mathcal{M}(|e_\theta\rangle\langle e_\theta|)\,) - \widetilde{D}_\alpha(\Gamma\||e_\theta\rangle\langle e_\theta|\,) &\le \frac{1}{\alpha-1}\log\left[\frac{1}{3^\alpha}\left(\left(\frac{2}{3}\right)^{1-\alpha} + \left(\frac{1}{3}\right)^{1-\alpha}\right)\right] + \frac{\alpha}{\alpha-1} \\
&= \frac{1}{\alpha-1}\log\left[\left(\frac{2}{3}\right)^{\alpha}\left(\left(\frac{2}{3}\right)^{1-\alpha} + \left(\frac{1}{3}\right)^{1-\alpha}\right)\right] \\
&= \frac{1}{\alpha-1}\log\left[\frac{2}{3} + 2^\alpha\,\frac{1}{3}\right] \\
&< 0\,.
\end{aligned} \tag{167}$$

For $\alpha > 1$, one has $\widetilde{D}_\alpha(\Gamma\|\mathcal{M}(|e_\theta\rangle\langle e_\theta|)\,) < \infty$ and $\widetilde{D}_\alpha(\Gamma\||e_\theta\rangle\langle e_\theta|) = \infty$, and and therefore the difference is $-\infty$.

Supplementary References

[1] P. Faist and R. Renner, Physical Review X **8**, 021011 (2018).
[2] N. Datta, IEEE Transactions on Information Theory **55**, 2816 (2009).
[3] J. Watrous, *The theory of quantum information* (Cambridge University Press, 2018).
[4] M. Horodecki and J. Oppenheim, Nature Communications **4**, 2059 (2013).
[5] R. F. Werner, Physical Review A **58**, 1827 (1998).
[6] G. Chiribella, in *Conference on Quantum Computation, Communication, and Cryptography* (Springer, 2010) pp. 9–25.
[7] G. Chiribella and Y. Yang, New Journal of Physics **16**, 063005 (2014).
[8] G. Chiribella, G. D'Ariano, P. Perinotti, and N. Cerf, Physical Review A **72**, 042336 (2005).
[9] A. Peres, Physical Review Letters **77**, 1413 (1996).
[10] P. Horodecki, Physics Letters A **232**, 333 (1997).
[11] M. Horodecki, P. W. Shor, and M. B. Ruskai, Reviews in Mathematical Physics **15**, 629 (2003).
[12] L. Del Rio, J. Åberg, R. Renner, O. Dahlsten, and V. Vedral, Nature **474**, 61 (2011).
[13] M. Tomamichel, R. Colbeck, and R. Renner, IEEE Transactions on information theory **56**, 4674 (2010).
[14] P. Faist, F. Dupuis, J. Oppenheim, and R. Renner, Nature Communications **6**, 7669 (2015).
[15] M. Tomamichel, *Quantum information processing with finite resources: mathematical foundations*, Vol. 5 (Springer, 2015).
[16] M. Müller-Lennert, F. Dupuis, O. Szehr, S. Fehr, and M. Tomamichel, Journal of Mathematical Physics **54**, 122203 (2013).
[17] M. M. Wilde, A. Winter, and D. Yang, Communications in Mathematical Physics **331**, 593 (2014).
[18] F. Brandao, M. Horodecki, N. Ng, J. Oppenheim, and S. Wehner, Proceedings of the National Academy of Sciences **112**, 3275 (2015).
[19] G. Chiribella, E. Aurell, and K. Życzkowski, Physical Review Research **3**, 033028 (2021).
[20] V. Bužek, M. Hillery, and F. Werner, Journal of Modern Optics **47**, 211 (2000).
[21] P. Horodecki, Physical Review A **68**, 052101 (2003).
[22] F. Buscemi, G. D'Ariano, P. Perinotti, and M. Sacchi, Physics Letters A **314**, 374 (2003).
[23] S. Beigi, Journal of Mathematical Physics **54**, 122202 (2013).
[24] R. L. Frank and E. H. Lieb, Journal of Mathematical Physics **54**, 122201 (2013).
